# The Dynamics of Flower Development in *Castanea sativa* Mill.

**DOI:** 10.3390/plants10081538

**Published:** 2021-07-27

**Authors:** Ana Teresa Alhinho, Miguel Jesus Nunes Ramos, Sofia Alves, Margarida Rocheta, Leonor Morais-Cecílio, José Gomes-Laranjo, Rómulo Sobral, Maria Manuela Ribeiro Costa

**Affiliations:** 1 BioISI—Biosystems & Integrative Sciences Institute (BioISI), Plant Functional Biology Centre, University of Minho, Campus de Gualtar, 4710-057 Braga, Portugal; b7682@bio.uminho.pt; 2 LEAF—Linking Landscape, Environment, Agriculture and Food—Institute of Agronomy, University of Lisbon, Tapada da Ajuda, 1359-017 Lisboa, Portugal; mramos@isa.ulisboa.pt (M.J.N.R.); salves@isa.ulisboa.pt (S.A.); rocheta@isa.ulisboa.pt (M.R.); lmorais@isa.ulisboa.pt (L.M.-C.); 3 CITAB—Centre for the Research and Technology of Agro-Environmental and Biological Sciences, Universidade of Trás os Montes and Alto Douro, Quinta de Prados, 5000-801 Vila Real, Portugal; jlaranjo@utad.pt

**Keywords:** *Castanea sativa*, Fagaceae, homeotic genes, ABCDE model, monoecy, unisexuality

## Abstract

The sweet chestnut tree (*Castanea sativa* Mill.) is one of the most significant Mediterranean tree species, being an important natural resource for the wood and fruit industries. It is a monoecious species, presenting unisexual male catkins and bisexual catkins, with the latter having distinct male and female flowers. Despite the importance of the sweet chestnut tree, little is known regarding the molecular mechanisms involved in the determination of sexual organ identity. Thus, the study of how the different flowers of *C. sativa* develop is fundamental to understand the reproductive success of this species and the impact of flower phenology on its productivity. In this study, a *C. sativa de novo* transcriptome was assembled and the homologous genes to those of the ABCDE model for floral organ identity were identified. Expression analysis showed that the *C. sativa* B- and C-class genes are differentially expressed in the male flowers and female flowers. Yeast two-hybrid analysis also suggested that changes in the canonical ABCDE protein–protein interactions may underlie the mechanisms necessary to the development of separate male and female flowers, as reported for the monoecious Fagaceae *Quercus suber*. The results here depicted constitute a step towards the understanding of the molecular mechanisms involved in unisexual flower development in *C. sativa*, also suggesting that the ABCDE model for flower organ identity may be molecularly conserved in the predominantly monoecious Fagaceae family.

## 1. Introduction

Development of unisexual male and female flowers has been regarded as a successful mechanism to decrease inbreeding by promoting cross-pollination and gene fluidity [1]. The development of unisexual flowers has evolved, independently, multiple times, in some families being the predominant reproductive strategy. Such is the case of the Fagaceae, a large plant family comprising more than 900 species that constitute an important economic resource and are pivotal to the dynamics of the forest ecosystems [2].

In the Fagaceae, the development of unisexual male and female flowers has been studied in *Quercus suber* (cork oak) [3,4,5]. Sobral and Costa [4] have shown that changes in the dynamics of the ABCDE model of flower organ identity may occur during the development of the male and female flowers of *Q. suber*. The canonical ABCDE model postulates that the development of the flower reproductive organs (stamens or carpels) is controlled by distinct classes of genes: stamen identity is determined by the interaction of B-, C-, and E-class transcription factors, whereas C-, D-, and E-class gene activity specify carpel identity [6,7,8,9,10,11]. The ABCDE genes belong, predominantly, to the highly conserved MADS-box family. These genes encode for transcription factors with four domains: MADS (M), intervening (I), keratin-like (K), and C-terminal (C) [12]. The MADS domain, which is the DNA-binding domain, is the most conserved, conferring these proteins the ability to bind to specific DNA motifs, the CArG-boxes [12,13]. The I and K domains are involved in the mediation and establishment of multimeric protein–protein complexes [14]. Although the C-terminal domain of the MADS-box proteins is much less conserved, it contains lineage specific motifs that are functionally important. In the specific case of B-class proteins, their stability and ability to bind CArG-boxes is dependent on an obligatory heterodimerisation [15,16,17,18]. In cork oak, the expression of one of the B-class genes, *QsPISTILLATA* (*QsPI*), determines the development of the male flower by establishing a complex with other B-class and C-class proteins. In female flowers, complete lack of *QsPI* expression disables the obligatory heterodimerisation of the B-class protein complex, thus promoting carpel development [4].

The male and female unisexual flowers of the Fagaceae species are typically unisexual by inception and develop at different periods during the growing season [4,19,20,21]. *Castanea* and *Lithocarpus* species present two distinct inflorescences, unisexual male catkins and bisexual catkins, the latter bearing functionally distinct male and female flowers [22,23] suggesting different mechanisms controlling the development of unisexual flowers within the Fagaceae. *Castanea sativa* Mill. is a deciduous Fagaceae species, distributed throughout the Mediterranean region, spreading from the Caucasus to Portugal and from Southern England to the southern part of the Iberian Peninsula [24]. It is monoecious, bearing two types of inflorescences: unisexual male catkins that develop in the leaf axis of the current season growth, and bisexual catkins that develop near the terminal end of the shoot. Each unisexual male catkin is composed by staminate flowers gathered in glomerules of 3 to 7 flowers each, in an average number of 40 glomerules per catkin [25]. Each individual male flower has four to six tepals and 8 to 12 stamens [26]. Female inflorescences appear singly or in two or three clusters at the base of the bisexual catkins. Each inflorescence usually has three flowers [26,27]. Each female flower typically presents six to eight styles, emerging from enclosed scales. The ovaries have five to seven loci with two viable ovules each, although usually only one of the ovules becomes a mature seed [23,27,28]. The stigmatic region, which is exposed during the receptivity period, is covered with secretory cells, which facilitates pollen adhesion and germination [26,28]. *C. sativa* presents a protandrous habit, in which unisexual catkins mature before the bisexual ones, and pollen from the unisexual catkin is released before the female flowers are fully receptive: this delay between pollen shedding and female flower maturation takes more than two weeks [26,29]. Cross-pollination is required to ensure fertilisation and fruit development due to gametophytic self-incompatibility and morphological male sterility of some cultivars [27,30]. 

Most genetic resources related to the *Castanea* genus are derived from studies performed in its close relative *Castanea mollissima* (Chinese chestnut), whose transcriptome and whole-genome have been recently released [31,32]. However, there is still no available transcriptomic data of *C. sativa,* despite its economic and ecological importance. Thus, the molecular mechanisms controlling the development of the *C. sativa* unisexual and bisexual catkins are still unclear, and it is not known to what extent these regulatory mechanisms are comparable to what has been described for other Fagaceae species. In the present study, we obtained a comprehensive transcriptome of *C. sativa* and studied the dynamics of BCE-class gene expression and BCE-class protein complexes during the establishment of the male and female flowers of *C. sativa*.

## 2. Results

### 2.1. Castanea sativa ‘Judia’ Cultivar Flowering Phenology

**Figure 1:**
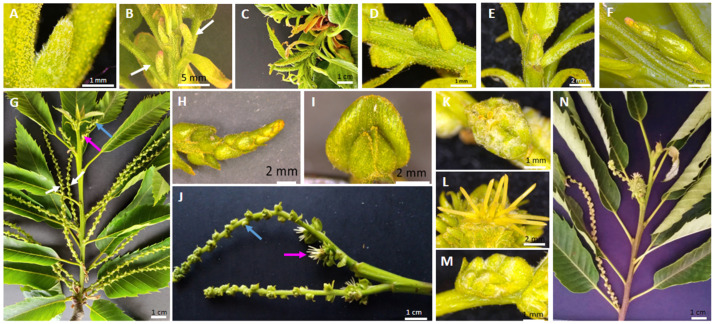
**Flowering phenology of *Castanea sativa*.** (**A**,**B**) In spring, following bud burst, the unisexual male catkins emerge in the axils of new leaves (white arrows). (**C**,**D**) As spring progresses, bracts start to open and expose the glomerules. (**E**,**F**) Bisexual catkin, with no clear distinction between the female and male flowers. (**G**) Shoot from June, in which the unisexual catkins reach their final stage of development (white arrows) and the differentiation between male (blue arrow, detailed in (**H**)) and female flowers (pink arrow, detailed in (**I**)) of the bisexual catkin is visible. (**H**) Detail of the male flower of the bisexual catkin. (**I**) Detail of the female flower of the bisexual catkin. (**J**) Developed bisexual catkin, with individualised male and female flowers (blue and pink arrows, respectively). (**K**) Glomerule of the unisexual male catkin, with anthers exposed. (**L**) Receptive female flower with exposed stigma. (**M**) Developed glomerule of the male flower of the bisexual catkin. (**N**) Shoot from July, in which the unisexual catkin is already turning yellow and dehiscent, having shed its pollen, and the female flower is swelling in order to develop the bur.

*Castanea sativa* is a monoecious species with an accentuated protandrous habit, as unisexual male catkins and bisexual catkins develop with a significant delay. In this study, a detailed phenological characterisation of the *C. sativa* ‘Judia’ cultivar was performed. The ‘Judia’ cultivar was chosen because it is a predominant cultivar in Portugal due to its high-quality fruits and productivity [30]. Unisexual male catkins emerge during spring in the axils of the new leaves that developed within the bursting axillary buds (Figure 1A,B). At this stage of development, the glomerules are still covered by bracts. As spring progresses, both the shoot and the unisexual male catkins elongate exposing the glomerules (Figure 1C,D). At the same time, bisexual catkins start developing at the terminal end of the new shoot, with no clear observable separation between female and male flowers (Figure 1E,F). The development of the female and male organs in the bisexual catkin is asynchronous as the male flower is the first to be identifiable. Towards the beginning of summer, the glomerules in the unisexual male catkins are fully exposed as the bracts start to recede, the anthers are visible, and pollen grains start to develop (Figure 1G, white arrow). During the same period, the separation between the male and female flowers becomes clearer in the bisexual catkin (Figure 1G, blue arrow, and detailed in Figure 1H (male flower); Figure 1G, pink arrow, and detailed in Figure 1I (female flower)). The styles of the female flowers are not receptive (Figure 1J, pink arrow) when the pollen from the unisexual male catkins is shed (Figure 1K). Thus, there is a temporal separation between pollen shedding of the unisexual male catkin and the period in which female flower is receptive (Figure 1L). The male flower in the bisexual catkin is not mature (Figure 1M) during female flower receptivity. During mid-summer and after pollen shedding, the unisexual male catkins start to acquire a yellow colour and fall. At this stage, pollen shedding occurs in the male flower of the bisexual catkin, while the female flowers have already been fertilised and start to swell (Figure 1N). 

### 2.2. Castanea sativa De Novo Transcriptome Analysis

Despite the *C. sativa* economic and ecological importance, it is still not clear which molecular mechanisms are employed to control the flower phenology in this species as no genomic/transcriptomic resources were available. Therefore, the assembly of a *de novo C. sativa* transcriptome was vital to lay the foundations for molecular studies in this species. To obtain the transcriptome of *C. sativa*, we isolated total RNA from male flowers from the unisexual catkin and male and female flowers from the bisexual catkin, leaves, and buds at different developmental stages during the plant life cycle. The RNA samples were pooled together and sequenced using Illumina technology to generate 2 × 150 bp paired-end reads. After trimming, 134,286,842 sequences were obtained and used for *de novo* transcriptome assembly. A pipeline for *de novo* transcriptome assembly and processing proposed by Chabikwa et al. [33] was implemented, resulting in 164,926 contigs (Table S1). To evaluate the reliability and quality of this assembly, we mapped the reads used to generate the *de novo* transcriptome back to the transcriptome using bowtie2 [34]. The alignment rate of the input RNA-Seq reads was 97.49%, whereas alignment with the *C. mollissima* genome yielded an overall alignment rate of 62.93%.

After redundancy removal, 32,871 transcripts were obtained, with an average length of 906 bp, an N50 value of 1230 bp, and a GC percentage of 44% (Table S1). BUSCO is a tool that quantitatively assesses the quality and coverage of a transcriptome on the basis of evolutionarily informed expectations of gene content from eukaryotic genomes [35]. In the *C. sativa* transcriptome, 94.1% of the BUSCOS had a complete representation, indication a high-quality assembly, with 86.67% single copy genes (Table S2). The 32,871 transcript sequences were uploaded to Blast2GO [36], and blastx was performed against the NCBI nr database with an E value cut off of 1e-3; 29,607 sequences had blast hits, and 3264 had no hits. GO annotation categorised the transcripts in different functional groups according to cellular components, biological process, and molecular function (Figure S1).

### 2.3. ABCDE MADS-Box Transcription Factors Were Conserved in Castanea sativa

In cork oak, a closely related Fagaceae, the identity of the female and male sexual organs is dependent on the spatial activity of the ABCDE MADS-box genes [4]. It is not clear, however, as to the way in which in other Fagaceae, such as *C. sativa*, the ABCDE model is involved in the determination of flower unisexuality.

To check if sexual organ identity development in sweet chestnut is controlled by MADS-box genes, we screened the *de novo* transcriptome for homologs of *Arabidopsis thaliana* or *Solanum lycopersicum* MADS-box proteins (*SlTM6* was used as a query because there is no *TM6* homolog in *A. thaliana*). The blast queries included homologs for genes belonging to A-class (*AtAPETALA1*), B-class (*AtPI, SlTM6*, and *AtAPETALA3*), C- and D-classes (*AtAGAMOUS*, *AtSHATTERPROOF1-2*, and *AtSEEDSTICK*), and E-class (*AtSEPALATTA1-4*). The phylogeny of the *C. sativa* ABCDE MADS-box proteins was inferred using homologs of other angiosperms, and, whenever possible, homologs from an ancestral angiosperm (*Amborella trichopoda*) and a gymnosperm (*Pinus radiata*). On the basis of multiple alignments, we were able to conclude that all the retrieved sequences were complete and displayed the four conserved domains (M-, I-, K-, and C-terminals) (Figure S2).

**Figure 2:**
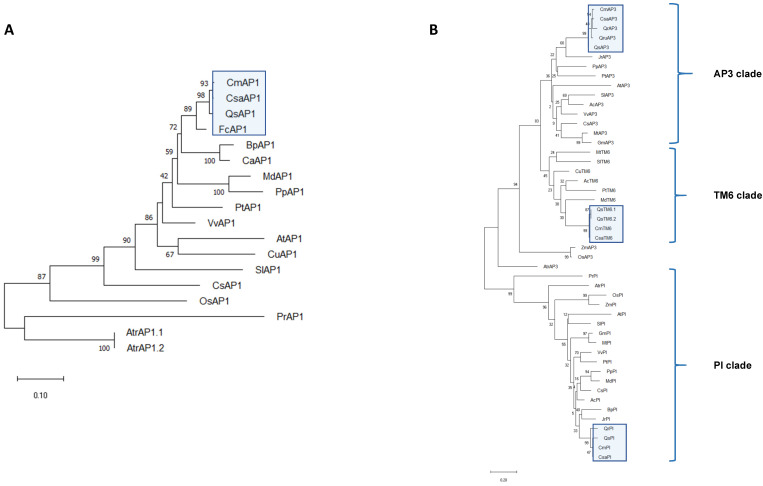

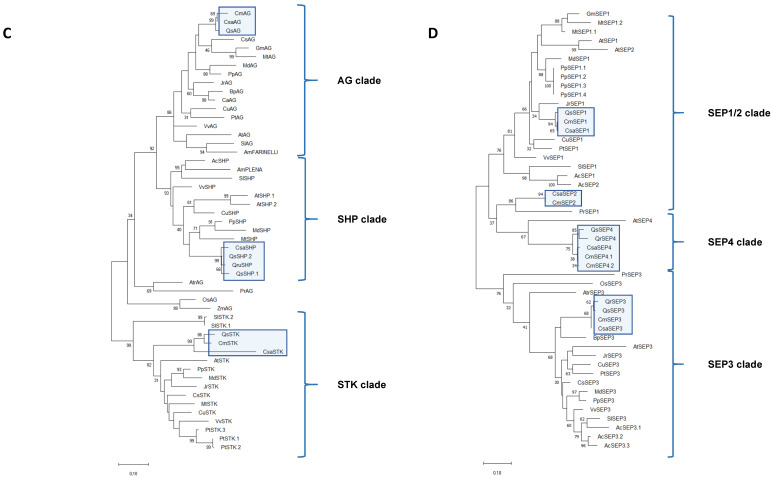
**Phylogenetic profiling of the *Castanea sativa* ABCDE genes.** (**A**) A-class lineage; (**B**) B-class lineage; (**C**) C- and D-class lineages; (**D**) E-class lineage. Phylogenies were inferred using the maximum likelihood method (1000 replicates). The percentage of replicate trees in which the associated taxa clustered together is shown next to the branch nodes. The JTT method was used to compute evolutionary distances (number of aminoacids per site). The accession numbers for the proteins used in this analysis are presented in Table S3. Fagaceae species are highlighted in the blue box. The protein sequences used in this analysis were retrieved from the following species: *Castanea mollissima* (Cm), *Quercus suber* (Qs), *Quercus robur* (Qr), *Quercus rubra* (Qru), *Fagus crenata* (Fc), *Betula pendula* (Bp), *Corylus avellana* (Ca), *Juglans regia* (Jr), *Malus domestica* (Md), *Prunus persica* (Pp), *Actinidia chinensis* (Ac), *Populus trichocarpa* (Pt), *Vitis vinifera* (Vv), *Arabidopsis thaliana* (At), *Citrus unshiu* (Cu), *Solanum lycopersicum* (Sl), *Cucumis sativus* (Cs), *Anthirrinum majus* (Am), *Medicago truncatula* (Mt), *Glycine max* (Gm), *Oryza sativa* (Os), *Zea mays* (Zm), *Pinus radiata* (Pr), *Amborella trichopoda* (Atr).

In the A-class (Figure 2A), CsaAPETALA1 (CsaAP1) clusters with AP1 proteins from other perennials, in the same clade that includes AP1-like proteins from *C. mollissima, Q. suber, Betula pendula*, and *Corylus avellana*, species belonging to the order Fagales. The B-class phylogroup (Figure 2B) can be divided into three lineages: PI, euAP3, and paleoAP3 [37]. The PI and paleoAP3 lineages arose from a duplication event that took place before the emergence of angiosperms, whereas a later duplication event that took place at the base of higher dicots originated the euAP3 lineage [37] (reviewed in [38]). One sweet chestnut homolog was identified in each lineage (CsaPI, CsaAP3, and CsaTM6) clustering with homologs of *C. mollissima, Quercus robur, Q. suber*, and *Quercus rubra*, all of them monoecious Fagaceae. The PI lineage is characterised by a PI motif that is essential for protein function [37]. A comparative analysis of the PI motif between AtPI and CsaPI revealed that CsaPI has a complete PI motif in its C-terminal domain that seems to be conserved in the Fagaceae (Figure S3A). The core difference between the paleoAP3 and euAP3 lineages lies in the motifs contained in the C-terminal domain (Figure S2B) [37]. Comparison of the euAP3 motif of *A. thaliana* and *C. sativa* homologs suggests a partial conservation of the euAP3 motif in CsaAP3, similar to what happens in other Fagaceae (Figure S3). Similarly, there is a partial conservation of the paleoAP3 motif in CsaTM6. This motif is totally conserved in *C. sativa* and *C. mollissima*, but there is one amino acid difference between proteins of the *Castanea* and *Quercus genus* (QsTM6) (Figure S3C).

The AG subfamily also underwent several duplication events throughout evolution: one duplication event originated the C and D lineages before the radiation of extant angiosperms [13]. The phylogenetic analysis for the C- and D-class proteins (Figure 2C) placed CsaAG in the same clade as *C. mollissima* and *Q. suber* AGAMOUS homologs. CsaSHP was also placed in the same clade as the homologs of the closely related Fagaceae *Q. suber* and *Q. rubra* (Figure 2C). There are two characteristic motifs (I and II) in the C-terminal domain of AG proteins [39]. CsaAG seems to have a partial conservation of these motifs, compared to its *A. thaliana* counterpart, and a complete amino acid conservation within the Fagaceae (Figure S2D). There is also partial conservation between CsaSHP and AtSHP AG motifs I and II, and a full conservation between the Fagaceae in motif I (Figure S2E). However, the same pattern is not followed regarding the AG motif II, where there is a complete deletion in QsSHP, which might suggest functional divergence.

The E-class comprises the *SEPALLATA* genes, which act as co-factors in the protein complexes that specify the identity of the different floral organs [40]. In the *C. sativa* transcriptome, four *SEP* homologs were identified (Figure 2D). Regarding the SEP1/2 lineage, both the CsaSEP1 and CsaSEP2 are closely related to the proteins of *C. mollissima* and *Q. suber*. CsaSEP3 also clusters with other Fagales; however, it is related to a closer degree with AtSEP3. CsaSEP4 is located in the same clade as SEP4 homologs of *C. mollissima, Q. suber,* and Q. *robur*. 

### 2.4. C. sativa BCE-Like Gene Expression in Unisexual and Bisexual Catkins

During floral developmental, the coordinated spatial and temporal regulation of the MADS-box gene expression is pivotal for proper floral organ development (reviewed in [40]). The canonical ABCDE model postulates that stamen identity is determined by the expression of B-, C-, and E-class genes in the third whorl, whereas carpel identity is determined by expression of C- and E-class genes in the centre floral whorl (reviewed in [13]).

An expression analysis, by qRT-PCR, of the sweet chestnut BCE-like gene homologs was conducted using samples that included male unisexual catkins, as well as male and female flowers from the bisexual catkins, at different stages of development. The developmental stages were collected after the emergence of the unisexual male catkin primordia until their full development (MF S1–S4, represented in Figure 1A–D, respectively); after the emergence of bisexual catkins (BP, Figure 1E–F), in which there is no observable separation between the male and female flowers, and after complete separation and developed male (MB 1, represented in Figure 1H, and MB 2, represented in Figure 1M) and female flowers (FB 1, represented in Figure 1I, and FB 2, represented in Figure 1L).

**Figure 3:**
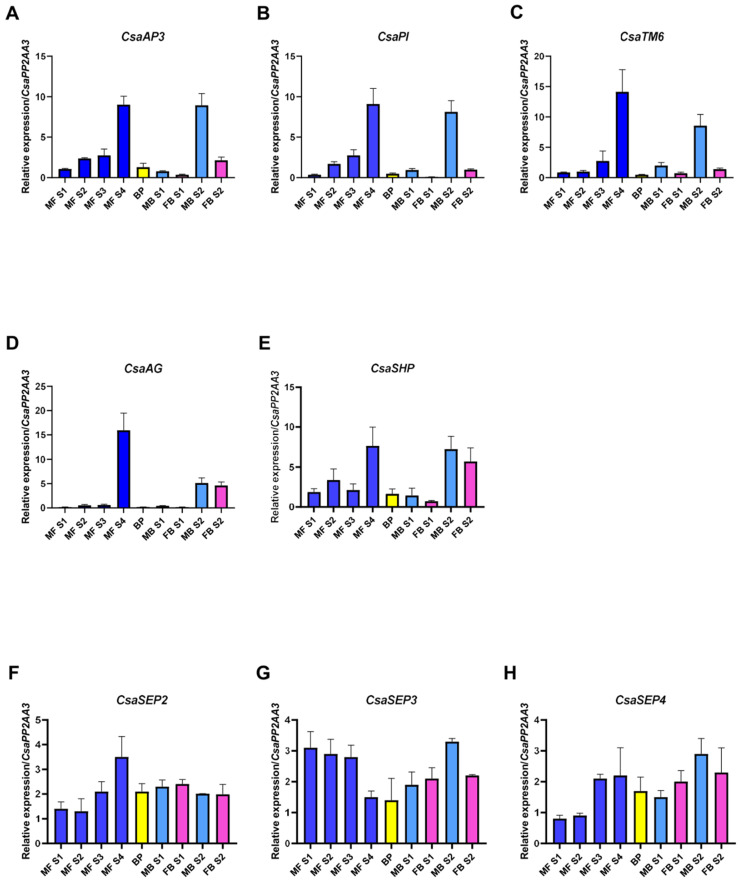
**Expression analysis of *C. sativa* BCE homolog genes in *Castanea sativa* flowers**. (**A**) *CsaAP3*; (**B**) *CsaPI*; (**C**) *CsaTM6*; (**D**) *CsaAG*; (**E**) *CsaSHP*; (**F**) *CsaSEP2*; (**G**) *CsaSEP3*; (**H**) *CsaSEP4*. Error bars indicate standard deviation (SD) of three biological and technical replicates. *CsaPP2AA3* was used as a reference gene. MF S1—unisexual male catkin primordia appearing in the axils of leaves of bursting buds (Figure 1A); MF S2—immature unisexual male catkins, starting to arise from bursting buds (Figure 1B); MF S3—mature unisexual male catkins (Figure 1C); MF S4—unisexual male catkin with developed glomerules, at a later stage of development (Figure 1D); BP—bisexual catkin primordia, with no definition between male and female flowers (Figure 1E,F); MB S1—male flowers of the bisexual catkin, early stage (Figure 1H); FB S1—female flower of the bisexual catkin, early stage (Figure 1I); MB S2—male flower of the bisexual catkin, fully developed (Figure 1M); FB S2—female flower of the bisexual catkin, fully developed (Figure 1L).

**Figure 4:**
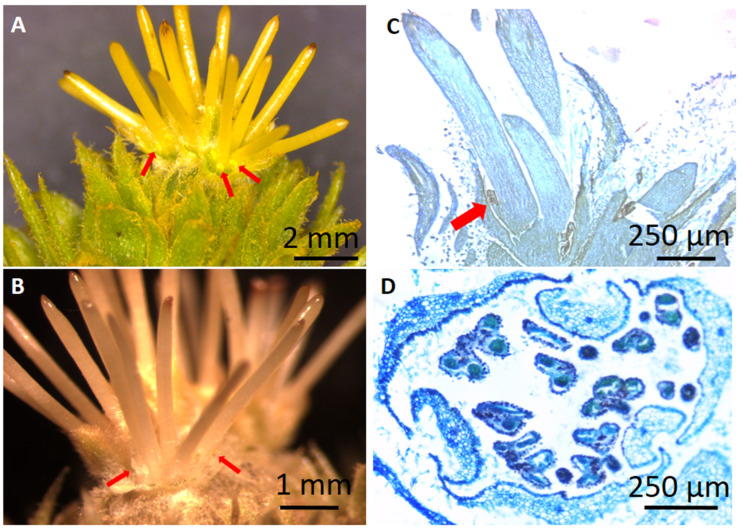
Female flowers from the ‘Judia’ cultivar present stamen-like structures. (**A**,**B**) Detail of the stamen-like structures at the base of the styles of female flowers—the perianth was removed to expose these organs (red arrows). (**C**) Longitudinal section of a female flower, where it is possible to see the stamen-like organ fused with the style (red arrow). (**D**) Transversal section of a male flower, with no evidence of stylar-like organs.

The B-class genes, *CsaAP3*, *CsaPI* and *CsaTM6* (Figure 3A–C), present similar expression patterns during the unisexual male flower development, increasing steadily from stages MF-S1 to MF-S4 (Figure 3A). The expression of *CsaAP3*, *CsaPI* and *CsaTM6* is low during bisexual catkin primordia development (BP) and in early stages of its male and female flowers development (MB-S1 and FB-S1), but the expression peaks in the male portion of the bisexual catkin in stage MB-S2 (Figure 3A–C). There is expression of *CsaPI* in the female structure of the bisexual catkin in stage FB-S2, although it is not detected in stage FB-S1 (Figure 3B). The expression of the three B-class genes in female flowers led to a more thorough morphologic and histological analysis of these flowers, which revealed the presence of stamen-like structures fused at the base of the styles, concealed by the perianth (Figure 4A–C). Histological analysis of the male flowers revealed that no carpelloid structures can be found in these flowers (Figure 4D).

*CsaSHP* expression follows a similar pattern to that of B-class genes, presenting low expression in early stages of male flower development and peaking at MF-S4 (Figure 3E). *CsaSHP* expression is low in bisexual primordia and in early stages after the separation of male and female structures, but increases in stages S2 for both tissues (MB-S2 and FB-S2), being higher in the male flowers than in the female flowers. The fact that *CsaSHP* is expressed also in the male flowers suggests that this gene might have retained a C-function after the duplication event that originated the C and D lineages. *CsaAG* is significantly more expressed in unisexual male flowers, mainly in the later stage (MF-S4), and in the bisexual catkins its expression follows a pattern similar to that of *CsaSHP*, with low expression levels in the early developmental stages, and higher in later stages of development (Figure 3D). *CsaSTK* expression was not detected in the flowers at the chosen developmental stages.

E-class genes *CsaSEP2* (Figure 3F) and *CsaSEP4* (Figure 3H) present low expression levels in early stages of unisexual male flower development, peaking in MF-S4. *CsaSEP3* (Figure 3G) shows an inverse expression pattern, being significantly higher during early development (MF S1) and decreasing in later stages. In the bisexual catkin primordia (BP) all the *SEP*-like genes are expressed, and upon early male and female flower development, their expression is higher in female flowers (FB S1) than in male flowers (MB S1). *CsaSEP1* was not detected in any of the flower tissues.

Overall, *CsaAG*, *CsaSHP*, *CsaAP3*, *CsaPI*, *CsaTM6* and *CsaSEP*s showed higher expression in the unisexual male catkin flowers and the male flower of the bisexual catkin than in female flowers, particularly at later stages of development.

### 2.5. Dimerisation of C. sativa BCE-Like Proteins

According to the canonical ABCDE model, stamen development depends on the interaction of B-, C-, and E-class proteins, whereas carpel development depends on the interaction of C- and E-class proteins [41,42]. Different dynamics of the ABCDE model in the Fagaceae *Q. suber*, namely, the interaction of B-class QsAP3 and QsPI with C-class QsSHP, and not QsAG (C-class), may be one of the factors underlying unisexual flower development in this species [4]. It is not clear if redeployment of the canonical ABCDE model interactions could be the mechanism behind the development of all the unisexual flowers in *C. sativa*.

**Figure 5:**
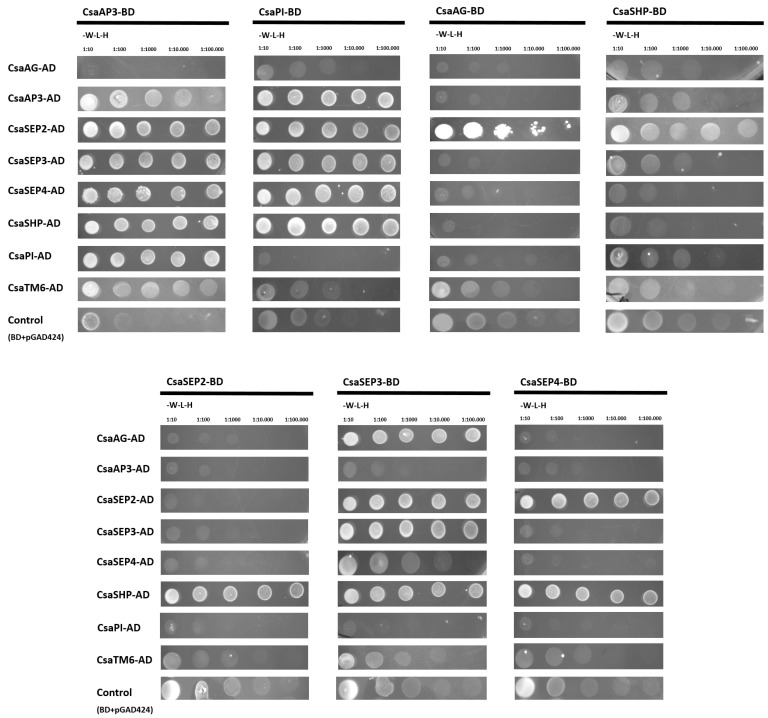
**Interaction between *Castanea sativa* BCE-like proteins.** Double yeast transformations of *C. sativa* BCE-like proteins fused to GAD4-activation domain (AD) and GAL4-binding domain (BD) were performed and tested for plasmid presence by growing cells in synthetic defined (SD) medium without tryptophan and lecucine (-W-L). Interacting capacity was tested in SD medium without aminoacids supplemented with 3-aminotriazole (tryptophan, leucine, histidine). Interaction strength was evaluated through sequential dilutions (1:10, 1:100, 1:1000, 1:10,000, 1:100,000). Each cropped image corresponds to a matching single plate in which the double transformants were selected.

Dimerisation of *C. sativa* BCE-like proteins was assessed in a yeast-two-hybrid experiment, in which the coding regions of the *C. sativa* B-, C-, and E-like genes were fused to the activation or binding domain of the GAL4 transcription factor. Interactions between *C. sativa* B-, C-, and E-like proteins are compiled in Figure 5.

An important trademark of the ABCDE model is the functional obligatory heterodimerisation between the B-class proteins. In *C. sativa*, the obligatory heterodimerisation of AP3 and PI-like proteins is likely to be conserved, as CsaPI and CsaAP3 proteins were able to dimerise and activate the reporter gene *HISTIDINE3*. CsaAP3 also interacted with its closest relative CsaTM6, but there was no interaction detected between CsaPI and CsaTM6. Interaction between B- and E-class proteins seems to be conserved in *C. sativa*, as all B-class proteins were able to interact with CsaSEP2, CsaSEP3, or CsaSEP4. CsaSEP1 protein was not used in this assay because *CsaSEP1* expression was not detected in flower tissues, as mentioned previously. The B-class proteins CsaPI and CsaAP3 were also able to interact with the C-class protein CsaSHP, but not with CsaAG (Figure 5).

The other C-class protein, CsaAG, was only able to interact with the E-class proteins CsaSEP2 and CsaSEP3, but the activation of the gene reporter was not sufficient to allow yeast growth in higher order dilutions, suggesting a weaker interaction between these proteins. The expression of CsSHP in male and female flowers combined with the non-canonical dimerisation of CsaSHP with B-class proteins suggests that CsaSHP might have retained a C-class function in the determination of floral reproductive organs.

## 3. Discussion

Changes to the ABCDE model dynamics is proposed as one of the factors controlling the development of unisexual flowers by inception in several families, including the predominantly monoecious Fagaceae [4,33,34,35]. In cork oak, several ABCDE gene homologs are differentially expressed in male and female flowers, suggesting that floral organ identity in this species is controlled by rearrangements in the expression dynamics of these genes. In the monoecious *C. sativa*, a similar scenario could also explain the development of its unisexual flowers. Therefore, it is of particular importance to characterise B- and C-function genes that specify male and female organ identity, in order to clarify the mechanism of sexual organ determination in *C. sativa*. In the present work, several genes homologous to the ABCDE model genes were identified and their role in the establishment of *C. sativa* unisexual flowers was assessed.

A *de novo* transcriptome of *C. sativa* was assembled and surveyed for ABCDE model genes homologs. According to this model, female flower identity is established by the coordinated activity of C- and E-class genes, whereas male flower development depends of B-, C-, and E-class genes [13]. In the present study, three B-class genes were identified and classified according to their phylogenetic clustering (*CsaAP3*, *CsaPI* and *CsaTM6*). B-class genes are expressed in the second and third whorl of the flower meristem, being involved in petal and stamen identity, respectively [13]. In *C. sativa*, the temporal expression profile for all B-class genes is similar, being higher in male flowers from the unisexual catkin and in the male flower of the bisexual catkin, and significantly lower in the female flower. 

**Figure 6:**
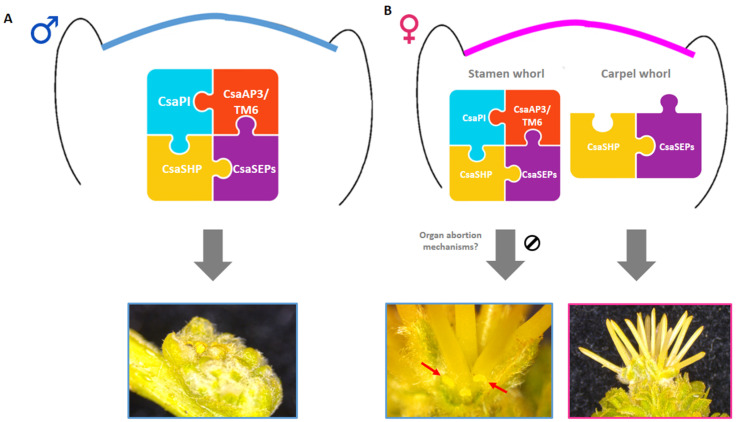
Representative model of the potential role of MADS-box transcription factors during the development of *Castanea sativa* unisexual flowers. (**A**) Combinatorial action of B-class CsaPI/TM6/AP3 with C-class CsaSHP and E-class CsaSEPs confers male identity to the unisexual male flowers. (**B**) Female identity is conferred by a combination of CsaSHP and CsaSEPs. Unknown mechanisms, downstream of the MADS-box genes, may contribute to the development of staminodes in female flowers (blue box, red arrows).

The expression pattern presented by the B-class genes *CsaTM6* and *CsaAP3* in *C. sativa* is similar to its close relative *Q. suber*: *QsTM6.1/2* and *QsAP3* present high expression levels in male flowers, being expressed in female flowers to a lesser extent [4]. However, this pattern is not mimicked by *CsaPI*, as there is expression of this gene in female flowers, in late developmental stages, which does not occur in *Q. suber*. Reports of hermaphroditism in *C. sativa* female flowers [26,36,37,38,39,40] suggest that the rudimentary stamens are sterile [27,36]. A pollen germination assay might be required to assess the fertility of these rudimentary stamens in the presently studied ‘Judia’ cultivar. The presence of these stamen-like organs in female flowers is not exclusive of *C. sativa*, having been also detected in other species of the *Castanea* genus. Morphologic observations in *C. mollissima* revealed the presence of 12 underdeveloped staminodes with short filaments and visible anthers capable of producing pollen, which did not dehisce [41,42]. In the Chinese chinquapin (*Castanea henryi*), female flowers experience a period of hermaphroditism, as 12 stamen primordia also develop during carpel development, arresting their elongation as the stigma enlarges [43]. It is thus likely that female flowers in the *Castanea* genus are hermaphroditic at inception and become unisexual by abortion or arrested growth of male organs. This is contrary to what was reported for other Fagaceae, in which the flowers are unisexual by inception as no aborted organs of the opposite sex are detected [4,19,20,21]. The expression of *CsaPI* and the other B- and C-class genes in the female flowers is likely associated to the development of the stamen-like structures in the female flowers of *C. sativa*. In the present study, CsaPI was found to be able to interact with CsaAP3 and CsaTM6, which is indicative that the obligatory heterodimerisation of B-class proteins is conserved also in this species. In the other Fagaceae *Q. suber*, this interaction was also shown [4]. Homodimerisation of AP3 homologs is also conserved in these two Fagaceae. However, the interaction between QsAP3 and QsTM6 was not reported for *Q. suber*, while in *C. sativa*, CsaAP3 and CsaTM6 were able to interact. Thus, it is likely that different interactions between Fagaceae B-class proteins might be species-specific. In *A. thaliana*, E-class genes have ubiquitous and redundant functions in flower meristem identity and flower organ development in the four whorls, as triple and quadruple mutants for these genes have floral organs converted into sepals and leaf-like organs [10,44]. In *C. sativa*, E-class proteins might have their function in flower organ identity conserved, as they are able to interact with B- and C-class proteins. Thus, similarly to what was determined in the close relative *Q. suber*, the heterodimerisation of B-class proteins and their interaction with C- and E-class proteins might be needed for male flower development in *C. sativa* (Figure 6).

In the *C. sativa* transcriptome, one AG protein (CsaAG) was inferred, which clustered in the euAG clade from the Fagaceae. In the PLENA lineage, one SHP-like protein was identified (CsaSHP). Expression analysis of these genes in *C. sativa* flowers revealed that *CsaAG* is mainly expressed in later developmental stages of male flowers, a pattern that has also been observed in other species with unisexual flowers, such as *Populus trichocarpa* [45], *Q. suber* [4], and *B. pendula* [46] with a strong association with pollen development. Unlike *CsaAG*, *CsaSHP* is expressed during unisexual male flower development, in the bisexual catkin primordia, and in the female and male flowers of the bisexual catkin. Similarly to *CsaSHP*, *QsSHP* was also detected in both the male and female flowers [4], suggesting that the Fagaceae *SHP* homologs, and not the *AG* homologs, could be acting as functional C-like genes.

Regarding the C-class proteins interactions, CsaAG only dimerises with CsaSEP3 and CsaSEP2. Similar results were found in *Q. suber* [4] and *Prunus mume* [47], in which AG-like proteins only interacted with SEP-like proteins. CsaSHP displayed interaction with all SEP-like proteins, which was also observed in species such as *Arabidopsis*, cork oak, and cucumber [4,48,49], indicative that the interactions between these proteins are relatively conserved among species. On the other hand, *C. sativa* B-class proteins CsaAP3 and CsaPI, but not CsaTM6, were also able to heterodimerise with C-class CsaSHP, as was previously reported for *Q. suber*, suggesting a conservation of this non-canonical interaction in the Fagaceae. The similar temporal expression patterns of *CsaAG* and *CsaSHP*, coupled with the fact that there was no observed interaction between B-class proteins and CsaAG, might indicate that *CsaSHP* may be required to act simultaneously with *CsaAG* in order to complete the C-function, as was observed in other species [4,50].

The temporal expression of the ABCDE model homologous genes in *C. sativa* and the ABCDE model interactome suggests that it is likely that the homeotic genes are involved in the determination of the male flower identity, both in the unisexual and bisexual catkins. However, the determination of unisexuality in the female flowers requires a different explanation. It is possible that the spatial expression of *CsaPI* in the female flower could be restricted to the non-reproductive and stamen whorls, allowing the development of stamens in the third whorl and the carpel in the centre whorl (Figure 6). Analysis of the spatial expression of *CsaPI* during the bisexual catkin primordia formation might help unveil the role of this gene during female flower development. Furthermore, the mechanisms that could be promoting the probable stamen abortion in this cultivar are still illusive. Thus, RNA-Seq studies concerning *C. sativa* inflorescences will be pivotal to clarify this hypothesis, as well as to identify genes exclusively expressed in female inflorescences that might be involved in the genetic basis of their development. It is possible that some of these genes are related to the developmental arrest of the stamens, as was reported in transcriptomic studies in other species in which sex determination is a late event [51,52,53,54].

Clarification of the mechanisms that lead to flower unisexuality are of particular interest in species of agronomical and ecological relevance, as it has biological implications, affecting fructification, seed production, and species perpetuation. *C. sativa* constitutes a particular case of variation of monoecy within the Fagaceae family, presenting unisexual male catkins and bisexual catkins. Furthermore, unlike most Fagaceae, *C. sativa* female flowers are not unisexual by inception. The likely temporary hermaphroditism in this species could be considered a relaxation that is counterbalanced by an additional layer to prevent inbreeding, which is the *C. sativa* gametophytic self-incompatibility. Thus, it is of utmost importance to study the genetic mechanisms for unisexual flower development in this species, which may differ significantly within the Fagaceae. The results here presented show that, even though phylogenetically close, ABCDE model genes may have different dynamics within distinct Fagaceae species.

## 4. Materials and Methods

### 4.1. De Novo Transcriptome Assembly

#### 4.1.1. Plant Material

Tissue samples (leaves, buds, and flowers in several developmental stages) from *Castanea sativa* M. were harvested at the germplasm bank located at UTAD (Vila Real district, Portugal, 41.2863826, −7.7448634) from three adult trees from the ‘Judia’ cultivar located in Lagoa (Vila Pouca de Aguiar district, Portugal, 41.6292217, −7.2960124). The flower developmental stages were selected on the basis of the phenological stages described in [55]. The collected tissues were frozen in liquid nitrogen and kept at −80 °C until RNA extraction.

#### 4.1.2. RNA Extraction

For *de novo* transcriptome assembly, total RNA was extracted from frozen tissue samples of young and mature leaves, dormant and active buds, and male and bisexual inflorescences in different stages of development. Samples were ground to powder using liquid nitrogen, and the RNA was extracted using the CTAB/LiCl method [56], with some modifications [57,58]. DNAse treatment was conducted using TURBO™ DNase (ThermoFischer, Waltham, MA, USA), following the manufacturer’s instructions, and integrity was analysed using the Experion™ RNA StdSens Analysis Kit (BioRad, Hercules, CA, USA), prior to library preparation.

#### 4.1.3. Library Preparation and Sequencing

The individual RNA samples were pooled, and the cDNA libraries were prepared using Illumina TruSeq stranded mRNA kit and sequenced at Fasteris S.A. (Plain-les-Ouates, Switzerland), using Illumina technology to generate 2 × 150 bp paired-end reads.

#### 4.1.4. *De Novo* Assembly of Castanea sativa Transcriptome

FastQC v0.11.8 [59] was applied to check the quality of the sequencing data. Low-quality reads were filtered and Illumina adapters removed using bbduk from the package bbtools [60]. All sequences with a quality score below 20 were removed and a forced trimming of the first 15 bp was performed. Overrepresented polyG sequences were removed using the option “literal”. Furthermore, in order to eliminate small reads that were interfering with the sequence length distribution, we filtered all reads with a length below 30 bp.

*De novo* transcriptome assembly was performed using Trinity (v. 2.9.1) [61,62] in a Galaxy server (https://usegalaxy.org/, accessed on 26 July 2021), with default settings. Assembly metrics were investigated using TransRate (v. 1.0.3) [63], a software that reports basic statistics such as assembly score, number of contigs, and N50. To examine the RNA-Seq read representation of the assembly, we mapped the reads used to generate the *de novo* assembly against the assembly using bowtie2 (v. 2.3.4.3) [64]. TransDecoder (Galaxy Version 3.0.1), with default settings, was used to predict coding regions within the transcriptome and remove redundancy. The single best open reading frame (ORF) per transcript longer than 100 peptides was used. CD-HIT-EST (v. 4.7) [65] was used with 95% similarity to reduce transcript redundancy and produce unique genes (henceforth “transcripts”). Benchmarking Universal Single-Copy Orthologs (BUSCO) [66], part of OmicsBox (v. 1.2.3, https://www.biobam.com/omicsbox, accessed on 26 July 2021), was used to validate the transcriptome assembly. Functional annotation of the whole *de novo* transcriptome was performed using Blast2GO (v. 5.2.5) [67]. The sequences were blasted against NCBI NR protein database (March, 2020) and were scanned with InterPro and mapped against the PSD, UniProt, Swiss-Prot, TrEMBL, RefSeq, GenPept, and PDB databases. The reads that originated the transcriptome were deposited in NCBI Sequence Read Archive under the accession number PRJNA744401.

### 4.2. Phylogenetic Analysis

*Castanea sativa* MADS-box transcript sequences were obtained using the blast+ software [68] by performing a BLAST in the *C. sativa de novo* transcriptome using *A. thaliana* sequences as query. Homologous protein sequences from other selected species were obtained by performing a BLASTx at the NCBI database (https://www.ncbi.nlm.nih.gov/, accessed on 26 July 2021), Hardwood Genomics (https://www.hardwoodgenomics.org/, accessed on 26 July 2021) and URGI (https://urgi.versailles.inra.fr/, accessed on 26 July 2021). Protein sequences were aligned using Clustal Omega [69], and distances were estimated using the Jones–Taylor–Thornton (JTT) model of evolution for a maximum likelihood tree with the MEGA X software [70]. A phylogenetic tree was generated from 1000 bootstrap datasets in order to provide statistical support for each node.

### 4.3. qRT-PCR Analysis

cDNA was synthesised using the Invitrogen cDNA synthesis kit SuperScript IV, according to manufacturer’s instructions. cDNA amplification was carried out using SsoFast EvaGreen Supermix (BioRad), 250 nM of each gene-specific primer, and 1 μL of 1/25 diluted cDNA. Quantitative real-time PCR (qRT-PCR) reactions were performed on the CFX96 Touch™ Real-Time PCR Detection System (Bio-Rad, Hercules, CA, USA). Gene expression analysis was based on three biological and three technical replicates, and was normalised with the reference gene *CsaPP2AA3* [71]. Following an initial period of 3 min/95 °C, each cycle (40 in total) consisted of a denaturation step of 10 s at 95 °C and annealing step of 10 s at specific primer temperature. Following the 40 cycles, a melting curve was obtained: the amplification products were heated in a gradient ranging from 65 to 95 °C in 5 s intervals. The primers used in the expression analysis are listed in Table S4.

### 4.4. Histological Analysis

For histological analysis, the flowers were collected and immediately fixed in 4% (w/v) paraformaldehyde in 1× PBS, under vacuum infiltration, followed by overnight incubation in the fixative solution at 4 °C. Samples were dehydrated, cleared, and embedded according to the protocol described by Coen et al. [72]. Tissue sections with 8 μm thickness were obtained using a microtome (SLEE) and mounted in pre-coated poly-L-lysine slides (VWR). The sections were deparaffined and stained according to Viejo et al. [73], with modifications.

### 4.5. Yeast-2-Hybrid Analysis

Protein–protein interactions were analysed using a GAL4-based yeast hybrid system (Matchmaker two-hybrid system; Clontech, Kusatsu, Shiga, Japan). Competent cells from *Saccharomyces cerevisiae* strain AH-109 were transformed with pGBT9 (bait vector; Clontech) and pGAD424 (prey plasmid; Clontech, Kusatsu, Shiga, Japan) derivatives, using the LiAc/DNA/PEG transformation method. Self-activation assays and selection of positive interactors were performed according to Causier and Davies [74]. To check for interaction, double transformations were selected first in SD medium without leucine and tryptophan and then in SD medium without leucine, tryptophan, and histidine supplemented with 3-aminotriazole. The primers used to construct the clones are listed in Table S4.

## Data Availability

The generated *de novo* reads used in this study are openly available in NCBI Sequence Read Archive under the accession number PRJNA744401.

## Supplementary Materials

The following are available online at https://www.mdpi.com/article/10.3390/plants10081538/s1, Figure S1: GO functional annotation of the Castanea sativa *de novo* transcriptome. Figure S2: Alignment of ABCDE-like amino acid sequences. Figure S3: Motif similarity in B- and C- MADS-box proteins between *C. sativa* and *A. thaliana*, *Q. suber* and *C. mollissima* (or *S. lycopersicum* in the case of TM6). Table S1: *De novo* assembly statistics of *Castanea sativa* transcriptome before and after redundancy removal. Table S2: *Castanea sativa* transcriptome completeness as determined by Benchmarking Universal Single-Copy Orthologous (BUSCO). Table S3: List of gene accessions. Table S4: List of primers.
