# Supplementary material for: The Dynamics of Flower Development in Castanea sativa Mill
Source: Plants (Basel). 2021 Jul 27;10(8):1538. doi: 10.3390/plants10081538 (PMC8398726; doi:10.3390/plants10081538)
Supplement: Supplementary file 1 [file plants-10-01538-s001.zip › plants-1311024-supplementary.pdf]

Table S1. *De novo* assembly statistics of *Castanea sativa* transcriptome before (Contigs) and after redundancy removal (Transcripts).

**Transcriptome assembly metrics**	
**Total number of raw reads**	193 896 450
**Total number of reads after trimming**	134 286 842
**Number of contigs in *de novo* transcriptome assembly**	164 926
**Number of transcripts in *de novo* transcriptome assembly**	32 871
**Average lenght of the transcripts**	906.25
**N50**	1 230
**GC %**	44

Table S2 – *Castanea sativa* transcriptome completeness as determined by Benchmarking Universal Single-Copy Orthologous (BUSCO)

**BUSCO notation**	**BUSCOs**
Complete Single-Copy	86.67%
Complete Duplicated	7.45%
Fragmented	3.53%
Missing	2.35%

Table S3 – List of gene accessions

A-Class	
**Given name**	**Protein name**
AtAP1	CAA78909.1
OsAP1	XP_015631033
PpAP1	XP_007223821.1
VvAP1	AAT07447.1
PtAP1	AAT39554.1
QsAP1	XP_023910685.1
MdAP1	ABG85297.1
SlAP1	NP_001234665.1
CmAP1	Cm_g17297.t1
BpAP1	CAA67967.1
CuAP1	A0A2H5NVU3_CITUN
CaAP1	Corav.3359
CsAP1	XP_011650331.1
AtrAP1.1	XP_006856356.1
AtrAP1.2	XP_020530401.1
PrAP1	AAB58907.1
FcAP1	BAP28174.1
**B-Class**	
**Given name**	**Protein name**
AtAP3	AAD51899.1
CmAP3	Cm_g20682.t1
VvAP3	NP_001267960.1
SlAP3	NP_001234077.2
MtAP3	XP_003603721.1
GmAP3	XP_014629918.1
CsAP3	NP_001295864.1
PpAP3	XP_020415740.1
AcAP3	PSS31295.1
PtAP3	XP_006386194.1
JrAP3	XP_018821033.1
ZmAP3	ACG37775.1
OsAP3	BAH22555.1
QsAP3	QSP122988.0
QrAP3	Qrob_P0454040.2
QruAP3	Quercus_rubra_120313_comp61142_c0_seq1_m.35060
AtrAP3	AAR06678.1
PrPI	AAF28863.1
AtrPI	XP_006847167.2
AtPI	P48007.1
VvPI	AAY79173.1
CsPI	NP_001292651.1
PpPI	XP_020410381.1

MdPI	CAC28022.1
AcPI	ADU15475.1
PtPI	XP_002300964.1
QsPI	XP_023887186.1
SlPI	ABG73411.1
BpPI	CAD32764.1
JrPI	XP_018811340.1
GmPI	XP_003523422.2
MtPI	ACJ36228.1
OsPI	AAC05723.1
ZmPI	ONM35875.1
CmPI	Cm_g4017.t1
QrPI	Qrob_P0600300.2
QsTM6.1	XP_023911257.1
QsTM6.2	XP_023911258.1
MdTM6	BAC11907.1
CuTM6	GAY57755.1
AcTM6	ADU15473.1
SlTM6	NP_001311309.1
PtTM6	XP_024461070.1
MtTM6	XP_003612101.1
CmTM6	maker-scaffold03851-augustus-gene-0.22-mRNA-1
**C/D-Class**	
**Given name**	**Protein name**
AtAG	NP_567569.3
AtrAG	NP_001292764.1
PrAG	AAD09342.1
AmPLENA	Q41195
AmFAR	Q9XFM8
SlAG	NP_001266181.1
CuAG	BAF34911.1
OsAG	XP_015632498.1
PtAG	XP_024455023.1
MdAG	XP_008383546.1
JrAG	XP_018830028.1
VvAG	NP_001268097.1
PpAG	XP_007211925.1
QsAG	QSP034737.0
BpAG	CAB95649.1
GmAG	NP_001237504.1
CmAG	AAZ77747.1
MtAG	XP_013462626.1
CsAG	NP_001292633.1
ZmAG	XP_008654205.1
AtrAG	NP_001292764.1
PrAG	AAD09342.1

AmFARINELLI	tr|Q9XFM8|Q9XFM8
AtSHP1	NP_001190130.1
QruSHP	Quercus_rubra_120313_comp19706_c0_seq1_m.
AcSHP	PSS16392.1
MtSHP	XP_024636027.1
SlSHP	NP_001300859.1
QsSHP1	QS001472.0
CmSHP	Cm_g3306.t1
AtSHP2	NP_850377.1
VvSHP	NP_001268105.1
MdSHP	CAC80857.1
PpSHP	XP_007217264.4
QsSHP2	QSP003163.0
CuSHP	BAF34914.1
AmPLENA	tr|Q41195|Q41195
QsSTK	XP_023895429.1
CmSTK	KAF3963586.1
JrSTK	XP_018841132.1
CsSTK	NP_001267506.1
CuSTK	GAY61853.1
PpSTK	ABQ85556.1
VvSTK	A0A217EJJ0.1
MdSTK	NP_001280931.1
MtSTK	XP_003598035.1
PtSTK1	XP_024439317.1
PtSTK2	XP_006376118.1
PtSTK3	XP_006371450.1
SlSTK1	XP_004241906.1
SlSTK2	XP_010322781.1
**E-Class**	
AtSEP1	NP_001119230.1
AtSEP2	AAU82009.1
AtSEP3	NP_564214.2
AtSEP4	NP_178466.1
GmSEP1	AAZ86071.1
MdSEP1	NP_001280893.1
MtSEP1.1	XP_024642411.1
MtSEP1.2	XP_024625956.1
PpSEP1.3	XP_007215876.1
MtSEP1.2	XP_024625956.1
PpSEP1.4	XP_007215877.1
PpSEP1.2	XP_020414319.1
PpSEP1.1	XP_020414318.1
CuSEP1	BAF95941.1
SlSEP1	AAM33104.2
QsSEP1	QSP061360.0

CmSEP1	Cm_g47769.t1
VvSEP1	NP_001268109.1
PtSEP1	XP_002306051.2
JrSEP1	Juglans_regia_01182017_WALNUT_00001177-RA_mRNA
AcSEP1	PSS35088.1
AcSEP2	PSS33641.1
CmSEP2	Cm_g360.t1
OsSEP3	XP_015648762.1
QsSEP3	XP_023899435.1
QrSEP3	Qrob_P0343740.2
CmSEP3	Cm_g11185.t1
BpSEP3	CAB95648.1
JrSEP3	XP_018836792.1
CuSEP3	BAF34912.1
PtSEP3	XP_024437662.1
CsSEP3	XP_004140534.1
MdSEP3	NP_001280756.1
PpSEP3	XP_007223808.1
VvSEP3	NP_001268114.4
SlSEP3	NP_001234384.1
AcSEP3.1	PSS13691.1
AcSEP3.2	PSS28600.1
AcSEP3.3	PSS26973.1
QsSEP4	QSP022774.0
CmSEP4.1	Cm_g17299.t2
CmSEP4.2	Cm_g17299.t1
AcSEP4	ADU15479.1
QrSEP4	Qrob_P0128890.2
AtrSEP3	NP_001292763.1
PrSEP1	AAD09206.1
PrSEP3	AAD09207.1

Table S4 – List of primers

**Amplicon**	**Direction**	**Sequence** (5' – 3')
CsaAP3 Y2H	Forward	AGGATCCAAATGGCAAGAGGAAAGATTCAGA
	Reverse	AACTGCAGCTACTCAAGCAAGGTGTAAGTTGTG
CsaPI Y2H	Forward	AGGATCCAAATGGGGAGAGGCAAGATTGAG
	Reverse	AACTGCAGTTACATTCTCTCTTGTAGATTTGGC
CsaTM6 Y2H	Forward	AAAGTCGACAAATGGGTCGTGGAAAGATCGAG
	Reverse	AACTGCAGTCAAGCAAGGCGCAGATCC
CsaAG Y2H	Forward	AGGATCCAAATGGTGTATCCGAACCAATCC
	Reverse	AACTGCAGTTAAACTAATTGAAGAGACATCTG
CsaSHP Y2H	Forward	AGGATCCAAATGGGAAGAGGAAAGGTAGA
	Reverse	AACTGCAGTTAAAGGACAACTCCATCTTGAA
CsaSEP2 Y2H	Forward	AGGATCCAAATGGGGAGAGGAAGAGTTGAGC
	Reverse	AACTGCAGCTAAAGCATCCACCCAGGAA
CsaSEP3 Y2H	Forward	AGGATCCAAATGGGTAGAGGAAGAGTGGAG
	Reverse	AACTGCAGTCATGGCATCCACCCTGA
CsaSEP4 Y2H	Forward	AGGATCCAAATGGGAAGGGGGAGAGTG
	Reverse	AACTGCAGTCAAAGCATCCAGCCTGGAAT
CsaAP3	Forward	TGGTGCTGTTATTGGATGCTCA
	Reverse	AGATCTGACCCTGCTCCACT
CsaPI	Forward	CCGAGAAATGCAGATGGAGT
	Reverse	AATAGGCTGCACACGGAAGG
CsaTM6	Forward	CTGCTCGATCTTAGGGCAAG
	Reverse	AGTTGGAGGCACCATTTGTC
CsaAG	Forward	CCAGCTTCTCCGAGCAAAGA
	Reverse	CATCTGGTCTTCACGTGGGT
CsaSHP	Forward	AGGGAAGTTGAGCTGCAAAA
	Reverse	CTGGGAGGTAGTTCCGATCA
CsaSEP2	Forward	CAGGCAGGTGACATTTGCTA
	Reverse	GCTGGCTTCCAATGCACTAT
CsaSEP3	Forward	TCAACACGGACGCAGTACAT
	Reverse	GGCCATATCCCATATCGTTG
CsaSEP4	Forward	GCCCTCATCATCTTCTCCAA
	Reverse	CCTCAACTTTTGCCTTCAGC
CsaPPA2A3	Forward	GGGTTCCCAACATCAAGTTC
	Reverse	TGACCTGATCACTTGACTGC

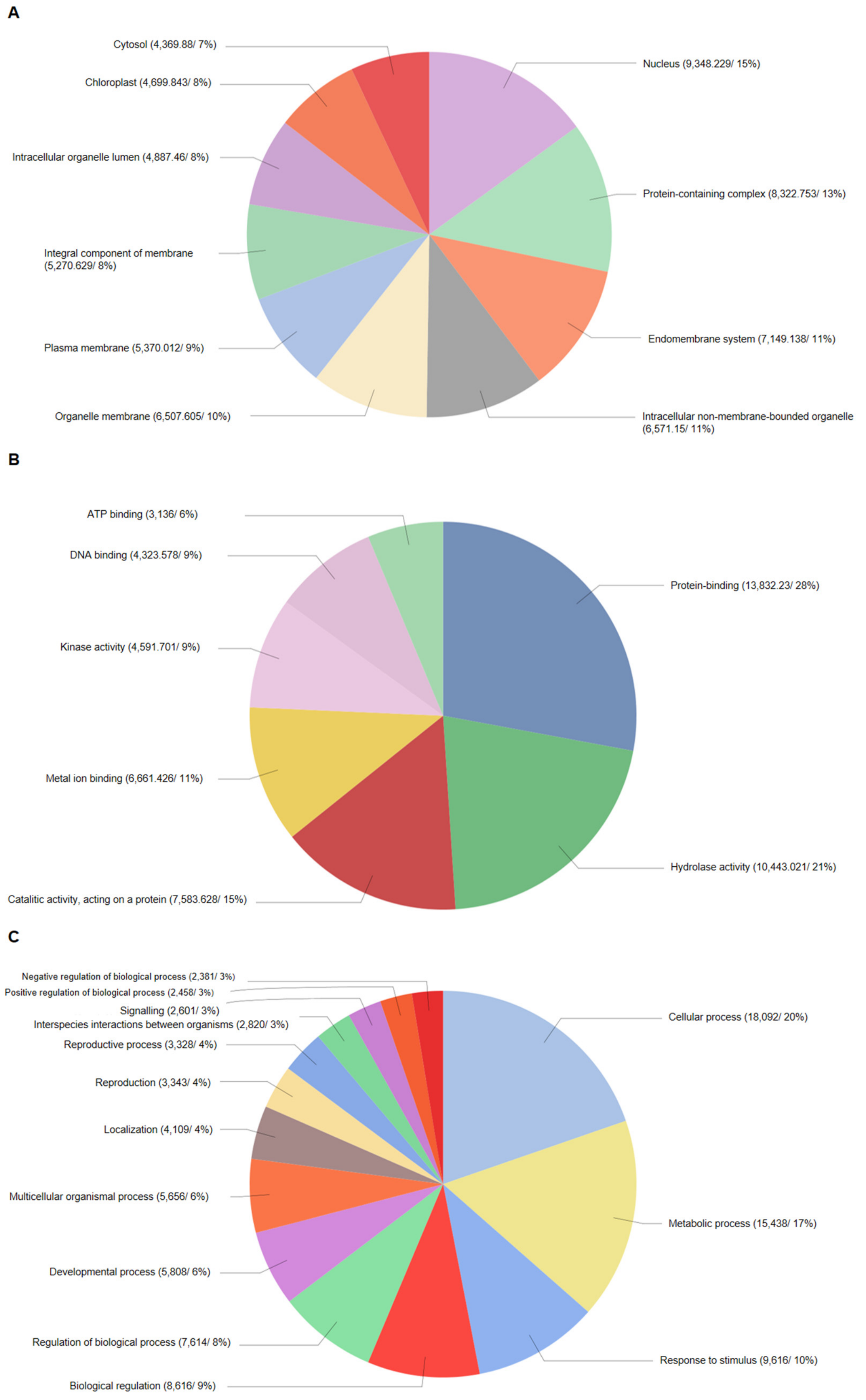

Figure S1 – GO functional annotation of the *Castanea sativa* de novo transcriptome. The transcripts were uploaded to Blast2GO and a blastx was performed, as well as an InterPro scan. The GO annotated transcripts were categorized according to cellular component **(A)**, biological process **(B)** and molecular function **(C)**.

A

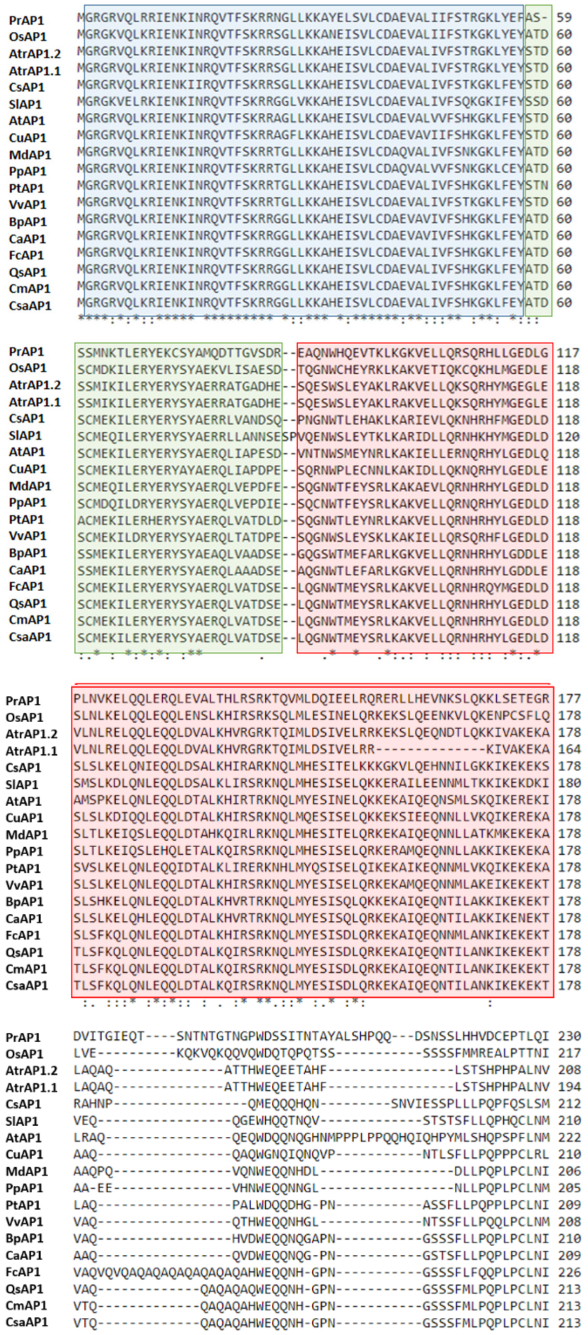
PrAP1
OsAP1
AtrAP1.2
AtrAP1.1
CsAP1
SlAP1
AtAP1
CuAP1
MdAP1
PpAP1
PtAP1
VvAP1
BpAP1
CaAP1
FcAP1
QsAP1
CmAP1
CsaAP1

B

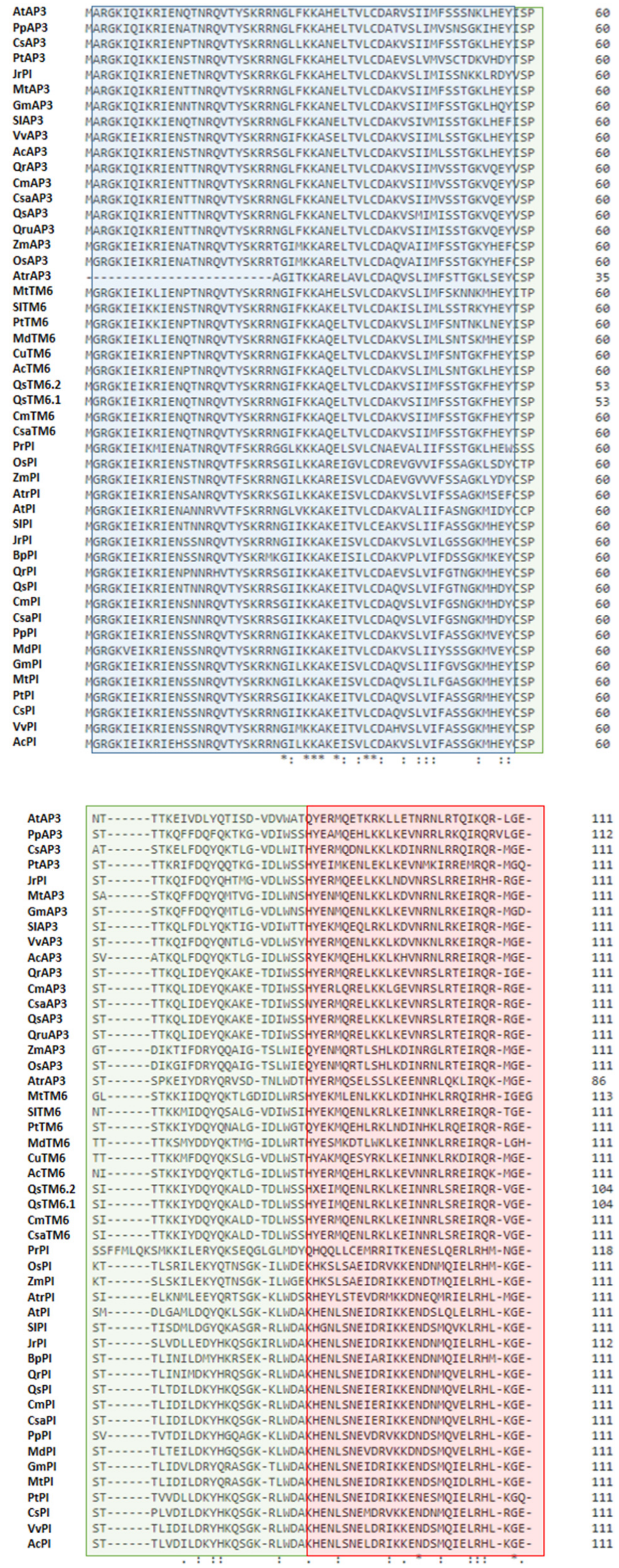
AtAP3
PpAP3
CsAP3
PtAP3
JrPI
MtAP3
GmAP3
SlAP3
VvAP3
AcAP3
QrAP3
CmAP3
CsaAP3
QsAP3
QruAP3
ZmAP3
OsAP3
AtrAP3
MtTM6
SlTM6
PtTM6
MdTM6
CuTM6
AcTM6
QsTM6.2
QsTM6.1
CmTM6
CsaTM6
PrPI
OsPI
ZmPI
AtrPI
AtPI
SlPI
JrPI
BpPI
QrPI
QsPI
CmPI
CsaPI
PpPI
MdPI
GmPI
MtPI
PtPI
CsPI
VvPI
AcPI

```
AtAP3    --CLDELDI-QELRRLEDEMENTFKLVRERKFKSLGSQIETTKKK---------------   153
PpAP3    --CLNDMSF-DELRGVEQEMEGAVEVIRKRKIRMISNQIDTTKKK---------------   154
CsAP3    --CMNDLSF-EELRCLEQDMDSAVRIIRERKYRVISNQIETHKKK---------------   153
PtAP3    --CLNGLSF-QDLQSLESDMESAWRVIHDRADRVLTNQIETSKKK---------------   153
JrPI     --SLIDLNL-EELYHLEQNVESAVKVIRDRKYHVIGKKTDILKKK---------------   153
MtAP3    --CLNDLSM-EELRLLEDEMDKAAKAIRERKYKVITNQIDTQRKK---------------   153
GmAP3    --CLNELGM-EDLKLLEEEMDKAAKVVRERKYKVITNQIDTQRKK---------------   153
SlAP3    --SLNDLNY-EQLEELMENVDNSLKLIRERKFKVIGNQIETYRKK---------------   153
VvAP3    --HLSDLSV-EELRDLEQEMESSLKMVRDRKYQVINNQIETFKKK---------------   153
AcAP3    --SLNELSY-EDLHGLELDVETSLKIIRDRNYKVISNQIATYKKK---------------   153
QrAP3    --SANDLSL-EKMHILEQDMENAVKVIRERKYRVISGQTETFKKK---------------   153
CmAP3    --SANDLSL-EKMHILEQDMENAVKVIRDRKYRVISNQTETFKKK---------------   153
CsaAP3   --SANDLSL-EKIHILEQDMENAVKVIRDRKYRVISNQTETFKKK---------------   153
QsAP3    --SANDLSL-EKMHILEQDMESAVKVIRDRKYQVINNQTQTFKKK---------------   153
QruAP3   --SANDLSL-EKMHILEQDMENAVKVIRDRKYRVISNQTVTFKKK---------------   153
ZmAP3    --DLDSLDF-DELRGLEQNVDAALKEVRHRKYHVISTQTDTYKKK---------------   153
OsAP3    --DLDGLEF-DELRGLEQNVDAALKEVRHRKYHVITTQTETYKKK---------------   153
AtrAP3   --ELNELRW-KDLRDLEQNLEEWVKRIRDKKNQLLTNQTDTCRKR---------------   128
MtTM6    GMELDDLSF-QQLRSLEEDMNSSIAKIRERKFHVIKTRTDTCRKK---------------   157
SlTM6    --DMSGLNL-QELCHLQENITESVAEIRERKYHVIKNQTDTCKKK---------------   153
PtTM6    --GLNDLSI-DHLRGLEQHMTEALNGVRGRKYHVIKTQNETYRKK---------------   153
MdTM6    --DLNGLSY-DDLRSLEDKMQSSLDAIRERKYHVIKTQTETTKKK---------------   153
CuTM6    --DLDDLTF-EELRGLEQNMSSSAATVRERKFHVIKTQTDTYKKK---------------   153
AcTM6    --ELNDLSV-HELRGLEQKMSASLTIIRDRKYHVIKTQTETYRKK---------------   153
QsTM6.2  --DLNGLSL-DDLRSLQNKVSSSLDIVRAR--------------K---------------   132
QsTM6.1  --DLNGLSL-DDLRSLQNKVSSSLDIVRARKYHVIKTQTETYKKK---------------   146
CmTM6    --DLNGLSL-DDLRSLQHKVSSSLDIVRARKYHVIKTQTETYKKK---------------   153
CsaTM6   --DLNGLSL-DDLRSLQHKVSSSLDIVRARKYHVIKTQTETYKKK---------------   153
PrPI     --EVNSLKL-PELFKLEEQLDKAATQVRRRKDHVLENERIKQRNK--MRRMEEENIILHG   173
OsPI     --DLNSLQP-KELIAIEEALNNGQANLRDKM----------------MDHW-----RMHK   147
ZmPI     --DLNSLQP-KDLIMIEEALDNGLTNLNEKLVTNPPSQSISMRLTLQMEHW-----ERRV   163
AtrPI    --DLNSLTP-HELNRIEDSLQMGLSSVRAKQ----------------MEHI-RTRTEMLK   151
AtPI     --DIQSLNL-KNLMAVEHAIEHGLDKVRDHQ----------------MEIL-----ISKR   147
SlPI     --DINQLTH-KELIIMEEALQNGLSSISAKQ----------------SEIL-----RMVR   147
JrPI     --DISSMNDAKELVLLEEALENGLSSIRERK----------------MDFL-----KMAK   149
BpPI     --DITSLNH-RELMILEEALQNGHSSIRERQ----------------MDLL-----MRAR   147
QrPI     --DITSLGP-KELILLEEALDNGLSSIRGKQ----------------------------A   140
QsPI     --DITSLNP-KELILLEEALENGLSSIREMQ----------------MEYL-----NIAT   147
CmPI     --DITSLNP-KELILLEEALDNGLSSIREKQ----------------MEYL-----NIAT   147
CsaPI    --DITSLNP-KELILLEEALDNGLSSIREKQ----------------MEYL-----NIAT   147
PpPI     --DITSLTH-KELMALENALENGLASNRDKQ----------------SKFV-----DMLR   147
MdPI     --DITSLNH-VELMALEEALENGLTSIRDKQ----------------SKFV-----DMMR   147
GmPI     --DITSLNY-KELMALEDALENGLSGVREKK----------------MEVH-----RMFK   147
MtPI     --DITSLNY-KELMALEESLENGLTGVRDKK----------------MEVH-----RMFK   147
PtPI     --DISSLPH-KELMAIEEALDTGLAAVRKKQ----------------MEFH-----SMLE   147
CsPI     --DITSLNY-KELMALEEALENGLTGVREKQ----------------SEFM-----KMMR   147
VvPI     --DISSLHH-KELMAIEDALEIGLASVRNKQ----------------MEFY-----KMVK   147
AcPI     --DITSLHH-KELMALEEALENGLSGIREKQ----------------MEVF-----KMMK   147
```

```
AtAP3    --NKSQQDIQKNLIHELEL-RAEDPHYGLVDNG--------GDYD----SVLGYQIE-GS   197
PpAP3    --LRSATEMNRNL-REFDA--RDDPHYGLVKNGR-------EDYE----SAFGYSSNGGP   198
CsAP3    --LKSVGEIHKSLLQEFDIATEEDPHYGLVDNGGVGVGIGGGDYE----SIMGFSGAAHP   207
PtAP3    --ARNVEQINRKLQVELEA-MDQDPY-GLVDNG--------GDYN----SVMGF------   191
JrPI     --KKRAESIHKVLLHELVA-RDEDPYGLVDNGG--------GDYC--DPDIIGCSNT-DS   199
MtAP3    --FNNEREVHNRLLRDLDA-RAEDPRFEMMDNG--------GEYE----SVIGFSNL-GP   197
GmAP3    --FNNEKEVHNRLLHDLDA-KAEDPRFALIDNG--------GEYE----SVIGFSNL-GP   197
SlAP3    --VRNVEEINRNLLLEFDA-RQEDPYGGLVEHD--------GDYN----SVLGFPTG-GP   197
VvAP3    --VRNVEQIHKNLLHEFDA-RDRDQYYGLVDNG--------GDYE----SVLGFSNG-SS   197
AcAP3    --LRNVEEIHRSLIHEFDA-IGEDSHYGLVDNE--------GDYD----SVLAYSNG-DH   197
QrAP3    --LRSAKDIHRNLLNGIDA-GNEDPQYGLVDNG--------GDYG----AVIGRSNG-DP   197
CmAP3    --LRNAKEIHRNLLHEIDA-GNEDPQYGLVDN---------GDYG----AVIGCSNG-DP   196
CsaAP3   --LRNAKEIHRNLLHEIDA-GNEDPQYGLVDN---------GDYG----AVIGCSNG-DP   196
QsAP3    --LRNAKEIHRNILHEIDA-GNEDPQYGLVDNG--------GDYG----AVIGCSNG-DP   197
QruAP3   --LRNAEQIHRNLLHEIDA-GNEDPQYGLVDNG--------GDYG----AVIGCSNG-DP   197
ZmAP3    --VKHSHEAYKNLQQELG--MREDPAFGYVDNT--------GAGVAWDGAAAALGGA-PP   200
OsAP3    --VKHSYEAYKTLQQELG--LREEPAFGFVDNT--------GGG--WDGGAG-AGAA-AA   197
AtrAP3   --INKLEAENNTIRLQME------------------------------------------   144
MtTM6    --VRSLEQMNGNLLLELEK-CVIHPQFLF-HDE--------GDEE----SAVALANG-AS   200
SlTM6    --ARNLEEQNGNLVLDLEAKCE-DPKYGVVENE--------GHYH----SAVAFANG-VH   197
PtTM6    --VKNLEERHGNLLMEYEAKLE-DRQYGLVDNE----------------AAVALANG-AS   193
MdTM6    --VKNLEERRGNMLHGYEAASE-NPQYCYVDNE--------GDYE----SALVLANG-AN   197
CuTM6    --VRNLEERHGNILLDFETKYD-DPHYGLVDN---------GDYQ----SAMALANG-AS   196
AcTM6    --VRNLEERHGNLLLDFEAKCDLAPQYELVENE--------GDYD----SAVAFANG-VS   198
QsTM6.2  --VKNLEERHGNLLLDLRARLE-DPQYGLVDNE--------GDYE----STIALTNG-AS   176
QsTM6.1  --VKNLEERHGNLLLDLRARLE-DPQYGLVDNE--------GDYE----STIALTNG-AS   190
CmTM6    --VKNLEERHGNLLLDLRARLE-DPQYGLVDNE--------GDYE----STIALTNG-AS   197
CsaTM6   --VKNLEERHGNLLLDLRARLE-DPQYGLVDNE--------GDYE----STIALTNG-AS   197
PrPI     MNQGHMEEDNGQFNFVLYQP-----VKKM----------------------R------TA   200
OsPI     RNEKMLEDEHKMLAFRVHQQ-----EVELSG---GIRELE-LGYH-HD--DRDFA---AS   192
ZmPI     TNTKMMEDENKLLAFKLHQQ-----DIALSG---SMRELE-LGYH-P---DRDLA---AQ   207
AtrPI    NNERILEDQNKQLKYIMHQI-----EGGDEAE---------RRYQ-NQQNGRDYP---QQ   193
AtPI     RNEKMMAEEQRQLTFQLQQQ-----EMAIASN---ARGMM----------MR-----DHD   184
SlPI     KNDQIXEEENKQLQYALHQK-----EMGAIGGSGNMRGIH-EEV--YHQRERDYE---YQ   196
JrPI     KNEKILEEENKHLNLILHQQ-----EIAMGTI---SRDVA-DDY--EQRVIRDYN---SQ   195
BpPI     KKDKILEEENKCLNFTLHQQ-----QMAMEAA--SCRDVE-DDY--EQR-VRDYN---SQ   193
QrPI     NGTEMLEEENKHLTLVLHQQ-----EMAMEA----NRDMN-DEY--QQR-VREYN---SQ   184
QsPI     RNTEMLEEENKHLALVLHQQ-----EMAMEA----NRDMN-DEY--QQR-VREYN---SQ   191
CmPI     KNTEMLEEENKHLTLVLHQQ-----EMAMEA----NRDMN-DEY--QLR-VREYN---SQ   191
CsaPI    KNTEMLEEENKHLTLVLHQQ-----EMAMEA----NRDMN-DEY--QLR-VREYN---SQ   191
PpPI     ENERALEEEHKRLTYELHK------QEMKIEE--NVRELE-NGY--RQR-LGNYN---NQ   192
MdPI     DNGKALEDENKRLTYELQKQ-----QEMKIEE--NVRNME-NGY--HQRQLGNYNNNQQQ   197
GmPI     RNDKILEEQNKELNFLLQQH-----LALEGVG--NMH----GQW--I-------------   181
MtPI     RNGKILEDENKELNFLLQQH-----MALEGVG--NMH----GQW--I-------------   181
PtPI     QNEKILDEEFKHLQFVLQQQ-----EMAMEE---NAMEME-NAY--HQQRVRDYN---SQ   193
CsPI     TNERMMEEENKRLNYELYQK-----EMVAMGD--SVREMD-IGY--NQR-MRDFN---SQ   193
VvPI     KNQRILEEENKHLNYIVHHQ-----GMPMEAG--NVREVE-SGY--HQRAVRDYN---PQ   194
AcPI     KNEKMLEDENKHLNFMLHQQ-----GMTMES-----REME-NGY--HQR-VRDYQ---HQ   190
```

C

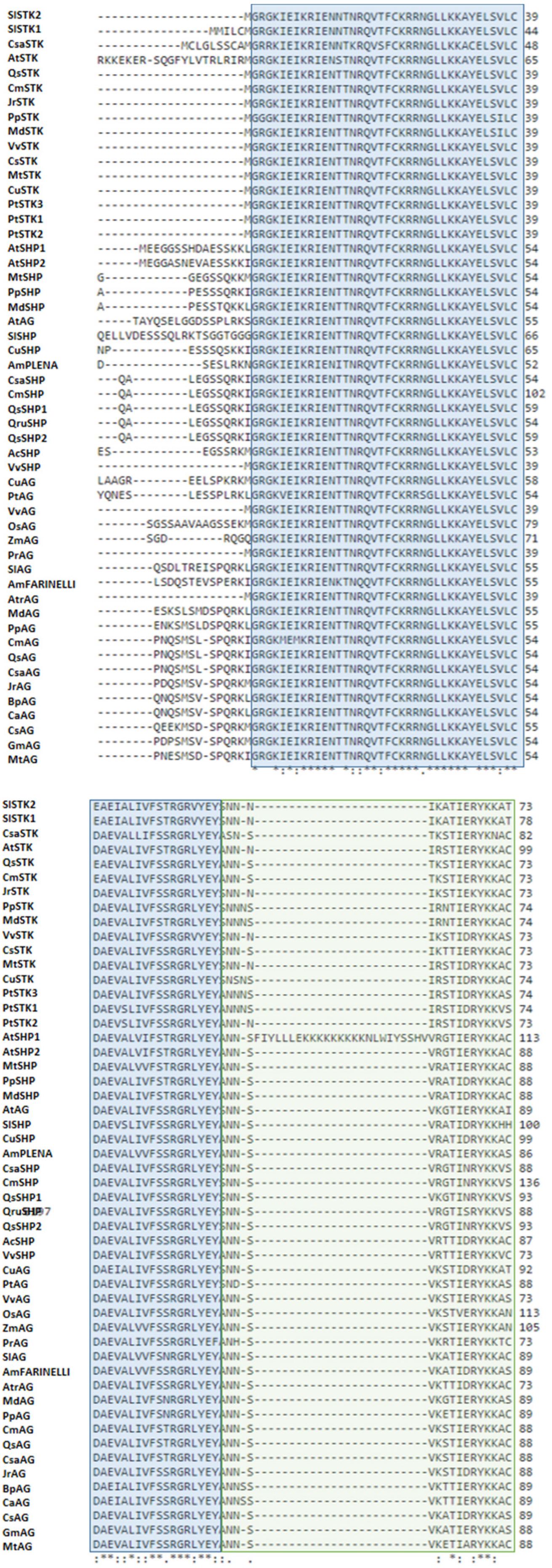

```
SlSTK2       AETSNACTTQELNA---QFYQQESKKLRQQIQMMQNSNRHLVGEGLSCLNVRELKQLENR 130
SlSTK1       AETSNACTTQELNA---QFYQQESKKLRQQIQMMQNSNRHLVGEGLSCLNVRELKQLENR 135
CsaSTK       LDSSETSSIAETNTQVNEYYQQESAKLRLLIQNMQDCNRHLLGEEVNSLNSKDLEQLESK 142
AtSTK        SDSTNTSTVQEINA---AYYQQESAKLRQQIQTIQNSNRNLMGDSLSSLSVKELKQVENR 156
QsSTK        NDGSGTSSIAQTNA---QYYQQESTKLRQLIQNMQNANRHLLGEGLSSLSIKEMKQLESR 130
CmSTK        NDDSGTSSTAQTNA---QYYQQESAKLRQLIQNMQNSNRHLLGEGLSSLNIKEMKQLESR 130
JrSTK        SDGSGTSSMAEVNA---QYYQQESAKLRQQIQMLQNSNRHLMGDALSSLSVKELKQLENR 130
PpSTK        SDSSGSTSITEINA---QYYQQESAKLRQQIQMLQNSNRHLMGDALSTLSVKELKQLENR 131
MdSTK        SDSTGSSSVTEINA---QYYQQESAKLRQQIQMLQNSNRHLMGDALSTLTVKELKQVENR 131
VvSTK        SDSTNGGSTMEINA---QYYQQESAKLRQQIQMLQNSNRHLMGDSLASLTVKELKQLENR 130
CsSTK        SDSSATSSVTELNT---QYYQQESAKLRQQIQMLQNSNRHLMGDSLSALTVKELKQLENR 130
MtSTK        SDHSSTTTTTEINA---QYYQQESAKLRQQIQMLQNSNRHLMGDALSTLTVKELKQLENR 130
CuSTK        SDNSNSGTVTEINA---QYYQQESAKLRQQIQMLQNSNRHLMGDSLSSLTVKELKQLENR 131
PtSTK3       SDSSNASSITEINA---QYYQQESAKLRQQIQMLQNSNRHLMGDAVSNLSVKELKQLENR 131
PtSTK1       SDSSNTASITEINA---QYYQQESAKMRQQIQLLQNSNRHLMGEAVSNLSVKELKQLENR 131
PtSTK2       SDSSNTASITEINA---QYYQQESAKMRQQIQLLQNSNRHLMGEAVSNLSVKELKQLENR 130
AtSHP1       SDAVNPPSVTEANT---QYYQQEASKLRRQIRDIQNSNRHIVGESLGSLNFKELKNLEGR 170
AtSHP2       SDAVNPPTITEANT---QYYQQEASKLRRQIRDIQNLNRHILGESLGSLNFKELKNLESR 145
MtSHP        AASTNAESVSEANT---QFYQQESSKLRRQIRDIQNLNRHILGEALGSLSLKELKNLEGR 145
PpSHP        TDSTNGGSVSEANT---QFYQQESSKLRRQIREIQNSNRHILGEALSTLNIKELKNLEGR 145
MdSHP        ADSTDGGSVSEANT---QFYQQEASKLRRQIREIQNSNRHILGESLSTLKVKELKNLEGR 145
AtAG         SDNSNTGSVAEINA---QYYQQESAKLRQQIISIQNSNRQLMGETIGSMSPKELRNLEGR 146
SlSHP        ADSTSTGSVSEANT---QYYQQEASKLRRQIRDIQTYNRQIVGEALGSLSPRDLKNLEGK 157
CuSHP        ADSSNPGSITEANT---QFYQQEATKLRRQIREIQNLNRHILGEALSTLNFKELKNLEAR 156
AmPLENA      ADSSNSVSTSEANT---QFYQQEANKLRRQIREIQTSNRQMLGEGVSNMALKDLKSTEAK 143
CsaSHP       IESSNPGTVAEVNA---QFYQQESSKLRRQIREIQNLNRHIVGEALSSMTFKELKNLEGR 145
CmSHP        TESSNPGTVAEVNA---QFYQQESSKLRRQIREIQNLNRHIVGEALSSMTFKELKNLEGR 193
QsSHP1       TESSNPGSVAEINA---QFYQQESSKLRRQIREIQNLNRHIVGEALSSMTFKELKNLEGR 150
QruSHP       TESSNPGSVAEINA---QFYQQESSKLRRQIREIQNLNRHIVGEALSSMTFKELKNLEGR 145
QsSHP2       TESSNPGSVAEINA---QFYQQESSKLRRQIREIQNLNRHIVGEALSSMTFKELKNLEGR 150
AcSHP        SDVLNTGSISESNT---QFYQQESNKLRRQIKDIQSSNKHILGEALSSLTFKELKNLEGR 144
VvSHP        SDSSNTGSVSEANA---QFYQQEASKLRRQIRDIQNLNRHILGEALSSLNFKELKNLETR 130
CuAG         ADTSNTGSICEANA---QFYQQEAAKLRIQISNMQNSNRNMLGESLSGLNFKELKNMETR 149
PtAG         ADSSNTGSVSEANA---QYYQQEAAKLRSQIGNLQNSNRHMLGEALSSLSVKELKSLEIR 145
VvAG         ADSSNTGSVSEANA---QFYQQESSKLHQQIRNLQNSNRHMLGESLGSLNFKDLKSLEIR 130
OsAG         SDTSNSGTVAEVNA---QHYQQESSKLRQQISSLQNANRTIVGDSINTMSLRDLKQVENR 170
ZmAG         SDSSNSGTVAEVNA---QYYQQESSKLRQMIHSLQNANTNIVGDSIHTMGLRDLKQMEGK 162
PrAG         VDNNHGGAISESNS---QYWQQEAGKLRQQIDILQNANRHLMGDGLTALNIKELKQLEVR 130
SlAG         SDSSNTGSVSEANA---QYYQQEASKLRAQIGNLMNQNRNMMGEALAGMKLKELKNLEQR 146
AmFARINELLI  SDSSLNGSISEANT---QYYQQEASKLRAQISNLQNQNRNMLGESLGALSLRELKNLESR 146
AtrAG        ADSSHSGTVSEANS---QYYQQEAAKLRNQIQVLTNTNRQLMGDSVGSMTVKELRTLENK 130
MdAG         ADSSNTGSVSEAST---QYYQQEAAKLRAQIVKLQNDNRNMMGDALSSMSVKDLKSLENK 146
PpAG         AESTNTGSVSEAST---QYYQQEAAKLRAQIGNLQNSSRHMMGESLSSMNMKDLKNLESK 146
CmAG         ADSSNTGSVAEANT---QFYQQESAKLRAQIGNLQNSKQANDGESLSNLTVKELKSLEIK 145
QsAG         ADSSNTGSVAEANT---QFYQQESAKLRVQIGNLQNSNRQMMGESLSNLTVKELKSLESK 145
CsaAG        ADSSNTGSVAEANT---QFYQQESAKLRAQIGNLQNSNRQMMGESLSNLTVKELKSLEIK 145
JrAG         ADSSNTGSVSEANT---QFYQREAATLRQQINSVQESNRKMLGESLSGMAFKDLKSLESK 145
BpAG         AESSNSGSVSEANT---QFYQQEAAKLRGQIRSVQNSNRHLLGEALSELNFKELKNLEIK 146
CaAG         ADSSNSGSVSEANT---QFYQQEAAKLRGQIRSVQDSNRHMLGEALSELNFKELKNLEKN 146
CsAG         SDSSNTGSTSEANT---QFYQQEAAKLRVQIGNLQNSNRNMLGESLSSLTAKDLKGLETK 146
GmAG         SDSSGAGSASEANA---QFYQQEADKLRQQISNLQNNNRQMMGDSLGSLTAKDLKNLETK 145
MtAG         SDSSGTGSATEANA---QFYQQEADKLRVQISNLQNNNRQMMGESLGSMNAKELKNLESK 145
                     :  : .:    .:*:*: .::  *  :    .     *: :  :  :::.  * .

SlSTK2       LERGISRIRSKKHEMILAETENLQKR--EILLEQENAFLRSKIAENERLQELSM----MP 184
SlSTK1       LERGISRIRSKKHEMILAETENLQKR--EILLEQENAFLRSKIAENERLQELSM----MP 189
CsaSTK       IVQGLVKIKSK--------------K--VMELENENAYLQAKVAEVERLKQANS-----N 181
AtSTK        LEKAISRIRSKKHELLLVEIENAQKR--EIELDNENIYLRTKVAEVERYQQHHH----QM 210
QsSTK        IEQGLTRIRSKKHEMLLAEIEYSQKR--VMELENESVYLQAKIAEVERLQQVNL-----N 183
CmSTK        IERGLTRIRSKKHEMLMAEIEYSQKR--VMELENESVYLQAKIAEVERLQQVNL-----N 183
JrSTK        LERGITRIRSKKHEMLLTDIEYLQKK--EIELENENICLRTKIAEVERLQQANM-----N 183
PpSTK        LERGINRIRSKKHEMLLAEIEYLQKK--EIELENENVCLRTKISEVERLQQANM------ 183
MdSTK        LERGITRIRSKKHELLLAEIEYFQKK--EIELENENVYLRTKVSEVERLQQANM------ 183
VvSTK        LERGITRIRSKKHELLLAEIEYLQKR--EIELENESVYLRTKIAEVERLQQANM------ 182
CsSTK        LERGITRIRSKKHEMLLAEIEYLQKR--EIELENENVCIRTKIAEVERVQQANM------ 182
MtSTK        LERGITRIRSKKHEMLLAEIEYFQKR--EIELENENLCLRTKINDVERLPQVNM------ 182
CuSTK        LERGITRIRSKKHEMLLAEIEFLQKR--EIELENESVCLRSKIAEMERFQQANM------ 183
PtSTK3       LERGITRIRSKKHELLLAEIEYLQKR--EIELENESVCLRTKIAEVERLQQANM------ 183
PtSTK1       LERGMTRIRSKKHELLLAEIEYMQKR--EIELENESACLRTKIAEVERLQQANM------ 183
PtSTK2       LERGMTRIRSKKHELLLAEIEYMQKR--EIELENESACLRTKIAEVERLQQANM------ 182
AtSHP1       LEKGISRVRSKKNELLVAEIEYMQKR--EMELQHNNMYLRAKIAEGARLNPDQQES--SV 226
AtSHP2       LEKGISRVRSKKHEMLVAEIEYMQKRVKEIELQNDNMYLRSKITERTGL--QQQES--SV 201
MtSHP        LEKGLSRVRSRKHETLFADVEFMQKR--EIELQNHNNYLRAKIAEHERAQQQQH----NL 199
PpSHP        LEKGISRIRSKKNEMLFAEIEFMQKR--EMELQNHNNYLRAKIAENERAQ-QQQ----TN 198
MdSHP        LEKGISRIRSKKNEILFSEIEFMQKR--ETELQHHNNFLRAKIAESEREQQQQQ----TH 199
AtAG         LERSITRIRSKKNELLFSEIDYMQKR--EVDLHNDNQILRAKIAENERNNPS-----ISL 199
SlSHP        LEKAIGRVRSKKNELLFSEIELMQKR--EIELQNANMYLRAKIAEVERAQEQ-----MNL 210
CuSHP        LEKGIGRVRSKKNEMLLAEIEFMEKR--EIQLQNDNMYLRARISENERAQQERQSESMMQ 214
AmPLENA      VEKAISRIRSKKNELLFAEIEHMQKR--ELELHNANMFLRAKIAEGERAQQQ-----MNL 196
CsaSHP       LEKGITRVRTKKNEMLLAEIEFMQKR--EVELQNENNYLRAKIAENERAQQEQG---TNF 200
CmSHP        LEKGIARVRTKKNEMLLAEIEFMQKR--EVELQNENNYLRAKIAENERAQQEQG---TNL 248
QsSHP1       LEKGITRVRTKKNEMLLAEIEFMQKR--EVELQNENNYLRAKIAENEXAQQQQG---TNL 205
QruSHP       LEKGITRVRTKKNEMLLAEIEFMQKR--EVELQNENNYLRAKIAENERAQQQQG---TNL 200
QsSHP2       LEKGITRVRTKKNEMLLAEIEFMQKR--EVELQNENNYLRAKIAENERAQQQQG---TNL 205
AcSHP        LEKAISRIRSKKNELLFAEIEHMQKR--EIELQNANMYLRAKIAENERAQQQ-----MNL 197
VvSHP        LEKGISRIRSKKNELLFAEIEYMQKR--EIELQNSNLFLRAQIAENERAQQQ-----MNL 183
CuAG         LEKGISRIRSKKNELLFAEIEYMQKR--EVDLHNSNQLLRAKIAENERGQQN-----MNL 202
PtAG         LEKGISRIRSKKNELLFAEIEYMQKR--EVDLHNNNQLLRAKISENERKRQS-----MNL 198
VvAG         LEKGISRIRSKKNELLFAEIEYMQKR--EIDLHNDNQYLRARIAENERNEQQ-----MSL 183
OsAG         LEKGIAKIRARKNELLYAEVEYMQKR--EVELQNDNMYLRSKVVENERGQQPLN--MMG- 225
ZmAG         LEKAIIKIRARKNELLYAEVDYMQKR--EMDLQTDNMYLRSKIAESNETGQPAMHMTMG- 219
PrAG         LEKGISRVRSKKNEMLLEEIDIMQRR--EHILIQENEILRSKIAECQNSHN------TNM 182
SlAG         IEKGISKIRSKKNELLFAEIEYMQKR--EVDLHNNNQYLRAKIAETERAQHQHQ--QMNL 202
AmFARINELLI  VERGISRIRSKKNELLFAEIEYMQKR-QEIDLHHNNQYLRAKIAESERVQG--Q--HMNL 201
AtrAG        LEKGISKIRSKKNELLFAEIDYMQNR--ELELQKDNMLLRAKIAENERAQH------MNM 182
MdAG         LEKAISRIRSKKNELLFAEIEYMQKR--ELDLHNNNQLLRAKIAENERGQQ-----NINV 199
PpAG         LEKGINRIRSKKNELLFAEIEYMQKR--EIDLHNNNQLLRAKIAENERSQQ-----NINV 199
CmAG         LERGISRIRSKKNELLFAEIEYMQKR--EVELHNNNQLLRAKIAENERNQQ-----NLNV 198
QsAG         LERGISKIRSKKNELLFAEIEYMQKR--EVELHNNNQLLRAKIAENERNQQ-----NLNV 198
CsaAG        LERGISRIRSKKNELLFAEIEYMQKR--EVELHNNNQLLRAKIAENERNQQ-----NLNV 198
JrAG         LESGIRRIRSKKNELLFAEIEYMQKR--EVDLHNNNQLLRAKIAENERNQQ-----NLNV 198
BpAG         LEKGINKIRSKKNELLFAEIEYMQKR--EAELHNNNQILRAKIAENERNQQ-----NLNV 199
CaAG         LEKGINRIRSKKNELLLAEIEYMHKR--EVDLHNNNQFLRAKIAENERNQQ-----NLNV 199
CsAG         LEKGISRIRSKKNELLFAEIEYMRKR--EIDLHNNNQMLRAKIAESERNVNMM------- 197
GmAG         LEKGISRIRSKKNELLFAEIEYMQKR--EIDLHNNNQLLRAKIAESERNHH-----NMAV 198
MtAG         LEKGISRIRSKKNELLFAEIEYMQKR--EIDLHNNNQLLRAKIAESERNHSHHSNINMSM 203
             :  .: :::::               :      *   .  ::::: :
```

D

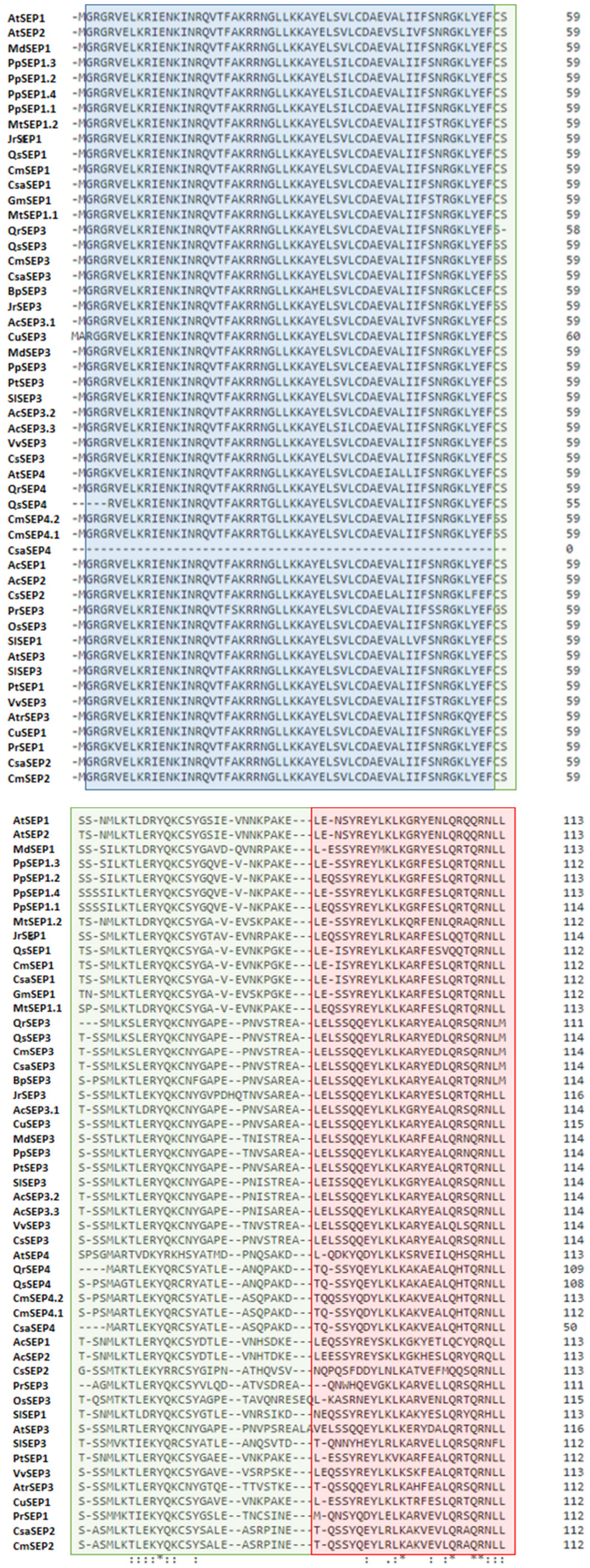

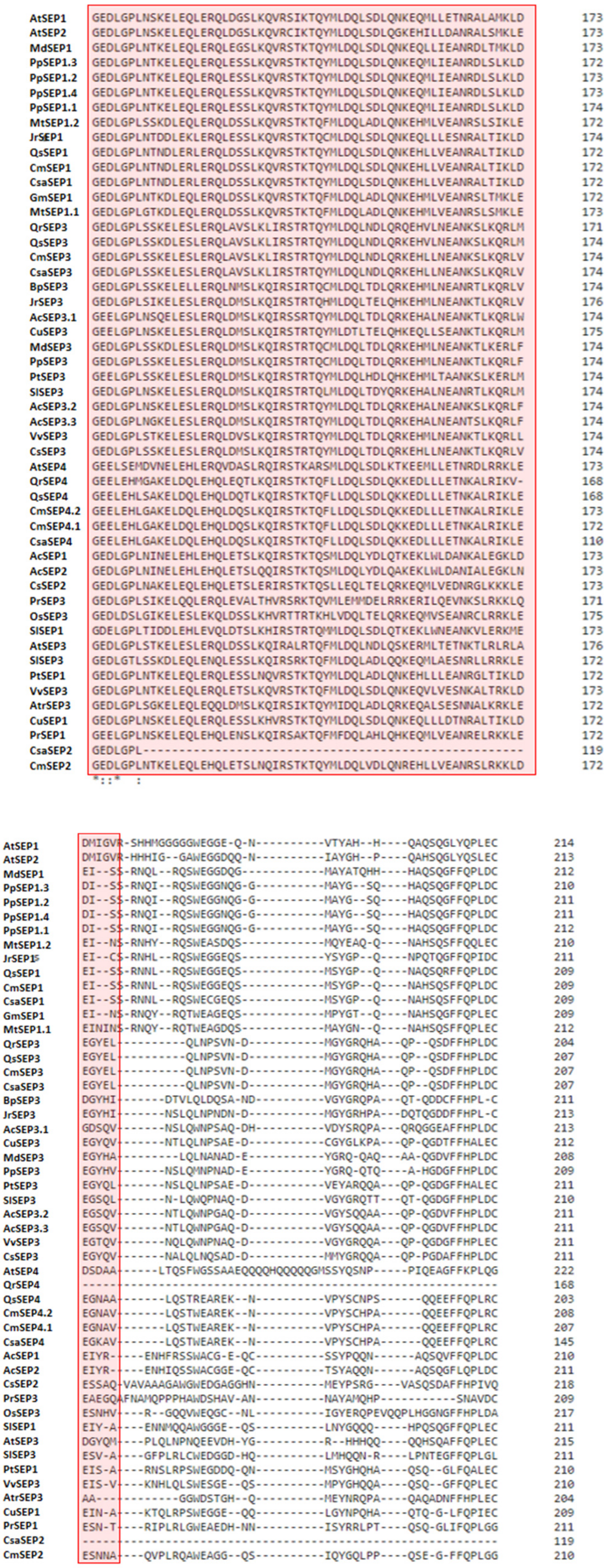

Figure S2 – Alignment of ABCDE-like amino acid sequences. Boxes indicate the conserved domains: MADS-domain (blue), I domain (green), and K domain (red). **A)** A-class proteins; **B)** B-class proteins; **C)** C and D-class proteins **D)** E-class proteins

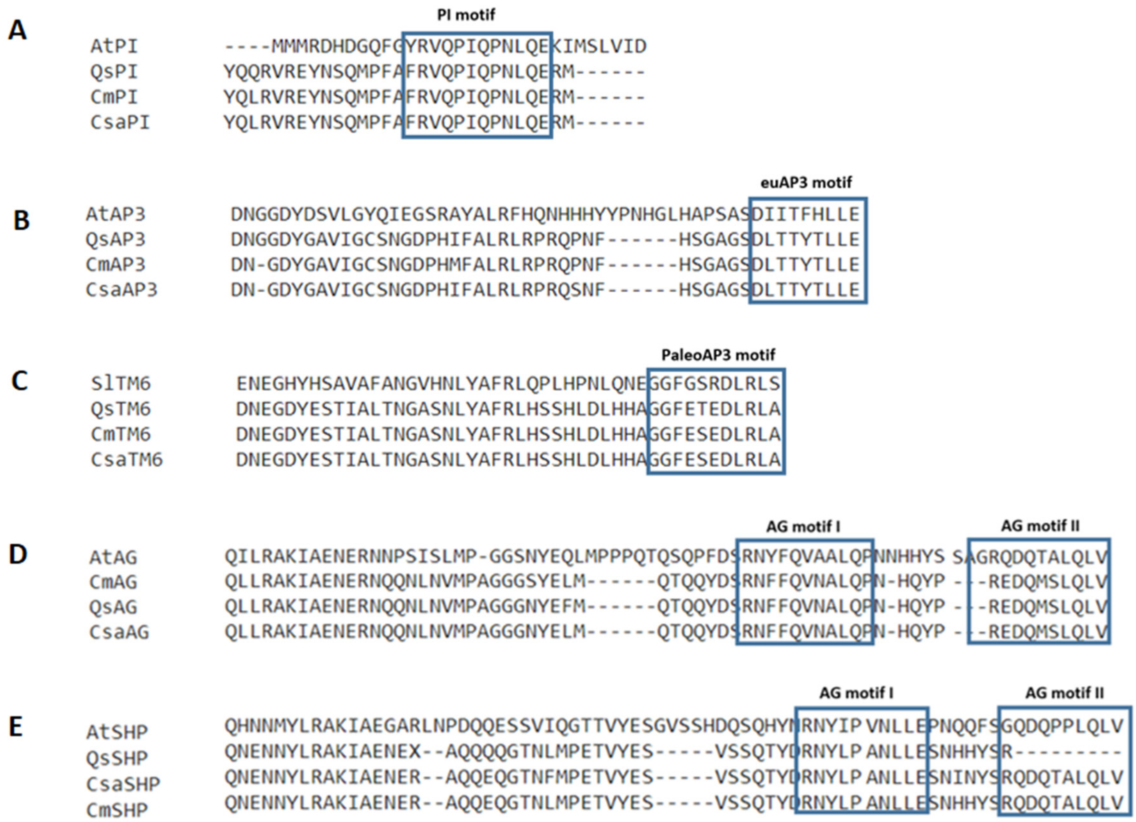

Figure S3 – Motif similarity in B and C MADS-box proteins between *C. sativa* and *A.thaliana*, *Q. suber* and *C. mollissima* (or *S. lycopersicum* in the case of TM6). **A)** PI-like proteins; **B)** AP3-like proteins; **C)** TM6-like proteins; **D)** AG-like proteins; **E)** SHP-like proteins
